# Soil water limitation intensity alters nitrogen cycling at the plant-soil interface in Scots pine mesocosms

**DOI:** 10.1007/s11104-025-07758-z

**Published:** 2025-08-26

**Authors:** Emily F. Solly, Astrid C. H. Jaeger, Matti Barthel, Johan Six, Ralf C. Mueller, Martin Hartmann

**Affiliations:** 1 Helmholtz Centre for Environmental Research – UFZ, Leipzig, Germany; 2 German Centre of Integrative Biodiversity Research (iDiv) Halle-Jena-Leipzig, Leipzig, Germany; 3 ETH Zurich, Zurich, Switzerland; 4 Research Institute of Organic Agriculture (FiBL), Frick, Switzerland

**Keywords:** Water limitation, Nitrogen cycling, Litter decomposition, ^15^N labeling, Metagenomics, Soil microbiome, *Pinus sylvestris*, Nitrogen allocation

## Abstract

**Background and aim:**

More intense episodes of drought are expected to affect terrestrial nitrogen (N) cycling by altering N transformation rates, the functioning of soil microorganisms, and plant N uptake. However, there is limited empirical evidence of how progressive water loss affects N cycling at the plant-soil interface.

**Methods:**

We adopted ^15^N tracing techniques and metagenomic analyzes of microbial genes involved in N cycling to assess how different levels of soil water availability influenced the fate of N derived from decomposing litter in mesocosms with Scots pine saplings.

**Results:**

With increasing water limitation, the release of N from decomposing litter into the soil declined rapidly. However, moderate levels of water limitation barely affected the microbial metagenome associated with N cycling and the uptake of N by the saplings. Comparatively, severe levels of water limitation impaired plant N uptake, and increased the prevalence of microbial N-cycling genes potentially involved in mechanisms that protect against water stress. Genes associated with the uptake and release of N during mineralization and nitrification declined under low soil water contents.

**Conclusions:**

When soil water becomes largely unavailable, the cycling of N at the plant-soil interface is slowed down, and microbial and plant tolerance mechanisms may prevail over N uptake and microbial decomposition.

**Supplementary Information:**

The online version contains supplementary material available at 10.1007/s11104-025-07758-z.

## Introduction

Soil water availability is a key factor influencing the physiological processes that determine plant growth and the functioning of soil microorganisms (Bose et al. 2022; Schimel 2018; Schuldt et al. 2020). The increasing frequency and intensity of droughts, as predicted by climate scenarios for the end of the twenty-first century, will lead to reductions in soil water availability, thereby affecting organic matter and nutrient cycling at the interface between plants and soils (Fuchslueger et al. 2024; IPCC 2021).

Nitrogen (N) is the primary limiting nutrient for plant growth in most non-fertilized terrestrial ecosystems (LeBauer and Treseder 2008; Reich et al. 2006; Tamm 2012), where litter decomposition is one of the main sources of bioavailable N in soils (Hobbie 1992). Reduced soil water availability can influence litter decomposition by decreasing the fragmentation and microbial mineralization of organic compounds, and limiting the leaching of soluble compounds into the soil (Krishna and Mohan 2017; Prescott 2010; Solly et al. 2014). However, the extent to which belowground and aboveground N cycling processes are altered likely depends on the severity of water limitation. The intensity of soil water deficits has been shown to influence the composition of the soil microbiome towards a reduction of known N-cycling taxa, and to impair tree growth and resource assimilation, relative to demand (Jaeger et al. 2024; McDowell et al. 2022; Solly et al. 2023).

During a period of drought, water limitation may profoundly alter the metabolism of the soil microbiome to help protect microorganisms from starvation and desiccation (Canarini et al. 2024; Gao et al. 2024; Malik et al. 2024). For instance, in dry soils, the diffusion of nutrient resources is constrained, and the osmotic stress increases, which may force microbes to accumulate N or produce osmolytes (Moyano et al. 2013; Schimel et al. 2007). An increased resource investment into maintenance pathways may in turn have an impact on other microbial processes such as the decomposition of organic N compounds, as well as the uptake and release of ammonium (NH_4_^+^) and nitrate (NO_3_^−^) during mineralization and nitrification processes. How environmental changes may affect the processes by which N resources are incorporated into soil microorganisms or are mineralized and rendered available to plants and the surrounding environment, is at the center of biogeochemical research (Neff et al. 2003; Stocker et al. 2025).

In addition to adverse impacts of water scarcity on N release from plant litter and the processes that partition N between plants and soil microorganisms, drought has been observed to rapidly affect tree N uptake and growth, with direct consequences on the N status of trees (Kreuzwieser and Gessler 2010). Periods of drought have been shown to impair fine root biomass, limiting N uptake in saplings of different tree species (Joseph et al. 2020). Moreover, studies on beech and oak saplings under conditions of water limitation have indicated an increase of osmoprotective amino acids at the expense of proteins involved in photosynthetic activity (Fotelli et al. 2002; Hu et al. 2013). Consequently, N uptake plays a crucial role in determining tree performance and its feedback to altered soil water levels due to environmental change.

Labeling techniques using ^15^N are frequently used to study N transformation and partitioning processes in forest soils. Most experiments have so far adopted the direct addition of labeled ammonium ^15^NH_4_^+^ or nitrate ^15^NO_3_^−^ to soils because the use of inorganic N allows a rapid investigation of the partitioning of N between soils and plants (Joseph et al. 2020; Walde et al. 2021). On the other hand, the application of ^15^N-labeled litter material, which facilitates the study of the cycling of N derived from the original litter within plant-soil systems, has more rarely been used because the production of labeled litter and the performance of experiments with multiple sampling time points are resource intensive (Bimüller et al. 2013). Alongside advances in isotopic labeling techniques, significant progress in microbial ecology has enabled the analysis of collective microbial genomes to provide insights into the metabolic potential of microbial communities (Myrold et al. 2014). Characterizing changes in the relative abundance of genes associated to different N cycling pathways and gene families can help enhance our knowledge of how the functional capacity of the soil microbiome to cycle N may be potentially affected by water limitation (Tu et al. 2018).

Even if the influence of water limitation on plants and soil microbial communities has been addressed in different contexts and experiments, there is still little mechanistic information available. Specifically, it remains unclear how different levels of prolonged water limitation influence the release of N from decomposing plant litter, the N metabolism of soil microorganisms, and the uptake and partitioning of N among aboveground and belowground plant tissues, over multiple time points across different seasons. Therefore, we conducted a ^15^N tracer experiment in a mesocosm platform with Scots pine saplings and natural forest soil to study how different levels of soil water contents affect the fate and cycling of N derived from decomposing needle litter at the plant-soil interface in the mesocosms. Moreover, we assessed changes in the soil metagenomic potential to cycle N in response to water limitation.

We hypothesized that reduced water availability would (i) decrease the amount of ^15^N label released from the added needle litter throughout the ^15^N tracer experiment, and thereby reduce the amount of ^15^N label recovered at the end of the study, (ii) increase the prevalence of N-cycling genes potentially involved in mechanisms protecting against water stress (e.g. those encoding for proteins and enzymes involved in the synthesis of osmoprotective and defense compounds), (iii) reduce the root biomass growth of the Scots pine saplings, and thus, the active surface area for N uptake. We were further interested in understanding whether the amount of incorporated ^15^N label in the saplings would be differentially allocated to aboveground and belowground tissues throughout the growing season under different levels of water limitation.

## Material and methods

### Experimental setup

The study was conducted in an experimental platform consisting of 18 Scots pine-soil systems (subsequently referred to as ‘mesocosms’). The setup of the mesocosms was conducted as previously described by Jaeger et al. (2023) and Solly et al. (2023), in two of the greenhouses of the Research Station for Plant Sciences (ETH Zurich, Lindau, Switzerland). Briefly, in September 2019 three-year-old Scots pine saplings (*Pinus sylvestris* L., seed origin Leuk, Switzerland, 980–1250 m a.s.l.) were planted in individual pots (32 cm height, 69 cm diameter, 100 L volume) filled with natural forest soil. The soil was collected from a mature xeric forest stand dominated by Scots pine trees in the Rhone Valley (Pfynwald, Canton Valais, Switzerland 46°18′16.1″N, 174 7°36′44.8″E, 600 m a.s.l). For three months the mesocosms were watered twice per week with 2 L of local rainwater reaching a volumetric water content (VWC) of approximately 30% (close to field capacity, which was ~ 35% VWC, with a pF of 1.8). In January 2020 the mesocosms started to be exposed to three different irrigation treatments in a randomized design to minimize spatial effects (i.e., variability in light availability). The three levels of irrigation were: sufficient water supply (control; 30% VWC; *n* = 6), decreased amount of water (intermediate; 40% reduction in VWC of control; *n* = 6), and water stress (severe; 75% reduction in VWC of control; *n* = 6). The intermediate water limitation treatment represents the maximum forecasted deviation of precipitation from the normal climate (1981–2010) for emission scenario RCP 8.5 in Southern Switzerland (NCCS 2018). The soil water content in the severe treatment was kept at a level at which the saplings received a minimum of water so as not to suffer from permanent damage and to maintain vitality. The mesocosms were equipped with soil sensors measuring VWC and soil temperature every 60 min (Teros 11, Teros 21, Meter Group, Pullman, WA, USA). The temperature in the greenhouses was regulated to account for seasonal changes in temperature according to climatological data measured at the MeteoSwiss meteorological station of Sion (Canton Valais, Switzerland), which is located close to where the forest soil used for the mesocosms was collected. Greenhouse temperatures and humidity were constantly monitored (Table S1). An automatic shading system prevented overheating of the greenhouses on hot and sunny days.

### Aboveground growth, needle fall, and leaf gas exchange of Scots pine saplings

Throughout the experiment, the height and stem diameter of the Scots pine saplings were monitored monthly as a proxy of aboveground incremental growth. The height was measured, including the buds. The diameter was measured at two angles, and the mean was taken. Here the monthly increment was calculated as radial growth. The needle fall was collected each season on a PE net (mesh size 3 mm × 3 mm) placed above the pot. The collected needle litter was dried for one week at 40 °C, weighed, and redistributed on the soil surface according to the average needle fall for each of the three irrigation treatments. At selected sampling dates, leaf gas exchange (light-saturated photosynthesis (Anet) and stomatal conductance (gs)) were measured using a LiCor 6400 system (LI-COR Biosciences, Lincoln, NE, USA), as detailed in Methods S1.

### ^15^N tracer experiment

With a ^15^N tracing experiment, we examined the influence of reduced water availability on the cycling of N derived from decomposing needle litter to the soil and into the Scots pine saplings.

Two types of intact Scots pine needles (hereafter referred to as ‘needle litter’) with differing N isotopic composition and similar N and C concentrations were experimentally prepared: natural abundance (1.5 ± 0.1 N %, 46.8 ± 0.1 C %, 0.366 ± 0.001 atom % ^15^N), and ^15^N enriched (1.5 ± 0.1 N %, 46.5 ± 0.1 C %, 21.955 ± 0.505 atom % ^15^N). The ^15^N enriched and the natural abundance needle litter were produced by growing two additional batches of Scots pine saplings of the same age and origin as those planted in the mesocosms. The two additional batches of Scots pine saplings were grown in individual pots (6 L volume filled with the same natural forest soil used in the mesocosms) and distinctively supplied with either a double-^15^N labeled ammonium nitrate solution (^15^NH_4_^15^NO_3_, ~ 85 atom % ^15^N) or with a natural abundance NH_4_NO_3_ solution (~ 0.36 atom % ^15^N) during the main growing season of the saplings (April to August 2020). In August 2020, the needles of the two additional batches of Scots pine saplings were harvested and oven-dried at 70 °C before being used for the ^15^N tracer experiment. A subsample of the needles of each sapling was milled and placed into tin capsules to measure the total N and C concentrations and N isotopic composition as described below.

Before applying the experimentally prepared needle litter to the mesocosms, three PVC rings (6 cm height, 6 cm diameter) were positioned at a distance of 20 cm around the stem of the saplings, and the old needle litter was removed from inside. The old needle litter was then replaced with the experimentally prepared new needle litter by adding 0.3 g of dry weight on the soil surface into each cylinder. The addition of experimentally prepared litter N at 0.0135 g N per mesocosm represented a maximum N addition of about 5% which can be considered a marginal disturbance of the N budget. The amount of N provided through litter fall every season was estimated at 0.26 to 0.33 g (Fig. S3, Jaeger et al. (2024)). The two types of experimentally obtained ^15^N enriched and natural abundance needle litter were each supplied to nine mesocosms in February 2021, and separated in two separate greenhouses to avoid cross-contamination after labeling with ^15^N (*n* = 3 per irrigation treatment and type of needle litter, Fig S1).

### Collection of plant and soil samples

Soil and plant samples were collected from each of the mesocosms before and after the start of the needle litter application for the ^15^N tracer experiment: in January 2021 (winter), May 2021 (spring), July 2021 (summer), September 2021 (autumn). This sampling time frequency was selected in order to capture most of the variability within the plant and soil system since seasonality has been shown to influence both plant metabolism and microbial communities (Gilson et al. 2014; Jaeger et al. 2023). At each sampling time point (season), 15 newly grown needles were collected from the saplings and immediately dried at 70°C. On the same day, bulk soil sampling was performed to a depth of ~ 20 cm with a slide-hammer corer (5.5 cm diameter). One individual soil sample per mesocosm for each sampling time point (season) was collected at a distance of ~ 10–15 cm from the cylinders where the needle litter was applied. The holes produced by the soil sampling were filled with sterilized sand to minimize soil structure disruption in the mesocosms. After sampling, the soil samples were kept cool, transported back to the laboratory, and stored at 4 °C. One day after the soil sample collection, the fresh soil samples were sieved to 4 mm. Roots were carefully picked out of the soil, and fine roots with a diameter < 2 mm were washed with Milli-Q water to remove any adhering soil particles. Dead roots were removed based on qualitative visual characteristics such as color and breakability (Solly et al. 2013). Living fine roots were dried at 70 °C and their dry weight was assessed.

### Soil properties

Fresh soil samples were used to measure gravimetric water content (GWC), ammonium (NH_4_^+^), and nitrate (NO_3_^−^) concentrations. A subsample (0.250 g of soil) for DNA extraction was stored at –20°C until further analysis. The rest of the soil samples were dried at 40 °C to constant weight and sieved to 2 mm for measurements of soil pH, total N, N isotopic composition, total C, and inorganic C. The GWC of the soil was assessed by weighing a subsample of 10 g of the soil before and after drying at 105 °C to constant weight. NH4^+^ and NO3^-^ concentrations of the soils were determined by extraction of 10 g soil with 50 ml 2M KCl solution in a 1:5 soil:solution ratio which was shaken for 1 h at 180 rpm. After filtering through a 150 nm ashless filter paper (Whatman No. 42), the extract was stored at −20°C until further analyzes. NH4^+^ and NO3^-^ concentrations were determined colorimetrically with a spectrophotometer v-1200 (VWR, Radnor, PA, United States) following Forster (1995) for NH4^+^ and Doane and Horwáth (2003) for NO_3_^−^. Soil pH was measured in a 1:2.5 solution containing 10 g of dried soil and 25 mL of 0.01 M CaCl_2_ solution. Samples were shaken horizontally at 180 rpm for 1 h and stored overnight to allow sedimentation before measurement with a pH meter (VWR, Radnor, PA, United States).

### Nitrogen isotopic composition of needles, fine roots, and soil

The dried needles, fine roots, and soil were milled, and weighed into tin capsules to measure the total N and C concentrations and N isotopic composition of the samples. Total N and C concentrations were determined with an elemental analyzer LECO 628 (LECO, St. Joseph, MI, United States). δ^15^N values were measured at the Stable Isotope Facility of the University of California, Davis (UC Davis, CA, USA) using a Sercon Europa 20–20 isotope ratio mass spectrometer (Sercon Ltd., Cheshire, United Kingdom). Soil inorganic C was measured with the pressure-calcimeter method following Sherrod et al. (2002). Concentrations of soil organic C were calculated as the difference between total and inorganic C.

### ^15^N mass balance calculation

The N isotopic composition is expressed in δ notation (‰) with the atmospheric standard AIR-N_2_ as reference. The ^15^N added by the ^15^N enriched needle litter in the different soil and Scots pine compartments (^*15*^*N excess, expressed as* μ*g g*^*−*^) over the course of the ^15^N tracer experiment, was calculated using the following equation (Eq. 1):

1 $${}^{15}N excess= \frac{atom\%{}{}^{15}Nl-atom\%{}{}^{15}Nn}{100} \bullet Npool$$

where *atom% Nl* describes the ^15^N/^14^N (in atom % ^15^N, Eq. 2) in a soil or Scots pine compartment at a given point in time after the start of the ^15^N tracer experiment, and *atom% Nn* is the ^15^N/^14^N in the natural background of the same compartment at the same point in time. The atom % ^15^N of the samples was calculated with Eq. 2:

2 $$atom\% {}{}^{15}N=\frac{100}{\frac{1}{\left(\frac{\delta }{1000}+1\right)*0.0036765}+1}$$

where 0.0036765 is the accepted N isotope ratio of AIR-N_2_.

The N pool (Eq. 3) was calculated as follows:

3 $$N pool=DW \bullet \frac{N\%}{100}$$

where *DW* represents the total dry weight of the Scots pine and soil compartments in the mesocosms (as detailed in Methods S2), and *N%* is the percentage of N in each compartment. The ^15^N excess of the Scots pine compartments (new needles, fine roots) was summed up for each sampling time point (season) to provide the total measured ^15^N label taken up by the saplings. Moreover, to calculate the relative fraction of ^15^N label allocated to the studied saplings compartments, the ^15^N excess of new needles and fine roots was divided by the total measured ^15^N label taken up by the saplings for each sampling time point (season). The ^15^N label derived from the decomposing needle litter recovered in the soil and Scots pine compartments during the course of the ^15^N tracer experiment was calculated by dividing the ^15^N excess different soil and Scots pine compartments by the quantity of ^15^N label added through the ^15^N enriched needle litter above the natural N isotope.

### DNA extraction and sequencing

DNA was extracted from 0.250 g of fresh soil, as described in Jaeger et al. (2023). Briefly, the extraction was performed using the DNeasy PowerSoil Pro Kit (Qiagen, Hilden, Germany) according to the manufacturer’s instructions using the QIACube System (Qiagen, Hilden, Germany). The quality and quantity of extracted DNA were measured via UV/VIS spectrophotometry with the QIAxpert System (Qiagen). Sequencing libraries were sequenced on Illumina NovaSeq 6000 (PE 150 bp, two S4 flow cells, or eight lanes) at the Functional Genomics Center Zurich (Zurich, Switzerland). The first sequencing revealed low coverage (sequencing yield) in 61 samples which were subsequently re-sequenced. These reads were merged with corresponding reads of the previous run after passing initial quality checks. The final yield (after merging) was 13.8 G fragments or 4.181 Tb. The average insert size was 245 bp (SD = 42 bp) as inferred by bbmerge.sh v.39.01 (BBMap 2022) with default settings on adapter-trimmed reads. The raw reads are deposited in NCBI's sequence read archive under the BioProject link: https://www.ncbi.nlm.nih.gov/bioproject/PRJNA1007885

All samples were processed individually (as detailed in Methods S3). Mapping statistics were extracted from annotated genes using the Ncyc database (Tu et al. 2018), and the result table for statistical analysis was produced for raw mapping counts and normalized counts based on transcripts per million (TPM). Details on the bioinformatics pipeline, including customized scripts and resources, are found here (10.5281/zenodo.15648671).

### Statistics

All statistical analyzes were computed in R Version 4.1.1 (R Core Team 2025). For all tests, a *p* < 0.05 was considered significant unless mentioned otherwise. To test the effect of the irrigation treatments and the sampling time points (seasons) on the investigated soil parameters (GWC, pH, NH_4_^+^, NO_3_^−^, total N, C: N ratio), and sapling parameters (height growth, diameter growth, needle litter fall, fine root biomass, new needle N, fine root N, soil N, soil C: N ratio, new needle N, new needle C: N ratio, fine root N, fine root C: N ratio), the data were fitted to linear mixed effect models. The effect of the irrigation treatments on the sapling A_net_, g_s_, and needle area was also assessed with linear mixed effect models. The *lme* function of the package *nlme* (Pinheiro J. 2022) was applied using the restricted maximum likelihood method “*REML*” (Meyer 1989). Whereas, irrigation treatment (control, intermediate, severe, *n* = 6) and sampling time point (winter, spring, summer, autumn, winter) were used as factor variables with interaction, the greenhouse (greenhouses 1 and 2) and the pot number (pots 1–18) were assumed to be a nested random effect. When visual inspection of diagnostic residual plots revealed that data deviated from the assumption of homoscedasticity or normality, the data were transformed using *log*_*10*_ or *sqrt*. Estimated marginal means with the *emmeans* function of the package *emmeans* were calculated to test the effects of the irrigation treatments and the sampling time points (seasons) on the ^15^N excess of the soil and Scots pine compartments, as well as the effect of sampling time points (seasons) on the percentage of ^15^N recovery and change in δ^15^N across the irrigation treatments.

Differences in the composition of microbial N-cycling genes among irrigation treatments and sampling time points (seasons) were calculated based on Bray–Curtis dissimilarities of square-root transformed TPM abundances and analyzed using permutational multivariate analysis of variance (PERMANOVA, Anderson (2001)) and permutational analysis of multivariate dispersion (PERMIDSP, (Anderson 2006)) implemented by the adonis2 and betadisper functions in vegan (Oksanen et al. 2025). Principal coordinate analysis (PCoA) was used to visualize differences in the composition of N-cycling genes under the different irrigation treatments and sampling time points (seasons). A correlation-based indicator species analysis using the *multipatt* function of the *indicspecies* package (Cáceres and Legendre 2009; De Cáceres et al. 2010) was performed to determine the association strength of all N cycling pathways (nitrification, denitrification, assimilatory nitrate reduction, dissimilatory nitrate reduction, N fixation, anammox, organic degradation, and synthesis), and of the gene families involved in the relatively most abundant pathway (organic degradation and synthesis), with the irrigation treatments. *P*-value adjustments for multiple comparisons were performed using the Benjamini–Hochberg method.

## Results

### Water limitation treatments

Following the start of the three irrigation treatments (control, intermediate, and severe), the VWC and GWC of the soils consistently differed among the treatments (Fig. S2, Table S2). The highest GWC values were observed under the control treatment, followed by the intermediate and severe water limitation treatments (Table S2, Table S3, *p* < 0.001, and as described in Jaeger et al. (2024)). Throughout all seasonal sampling points, the soil of the control mesocosms had an average GWC ranging between 12.3% and 33.9%. Soils of the mesocosms treated with intermediate and severe water limitation had an average GWC ranging from 3.8% to 13.8% and 2.2% to 4.7%, respectively. During the ^15^N tracer experiment, the highest soil GWC values were measured in winter and the lowest were observed in spring and summer for all treatments (Table S2, Table S3, *p* < 0.001).

### Effects of water limitation on Scots pine saplings

At the start of the ^15^N experiment (i.e. at the start of the second growing season of the saplings in the mesocosms), all saplings presented a similar height (73 ± 3 cm under the control treatment, 72 ± 3 cm under the intermediate water limitation treatment and 72 ± 4 cm under the severe water limitation treatment), and similar stem diameter (24.8 ± 1.6 mm under the control treatment, 21.9 ± 1.1 mm under the intermediate water limitation treatment and 23.5 ± 1.2 mm under the severe water limitation treatment). Nevertheless, over the course of the ^15^N experiment, the control saplings presented the largest growth increment in both height and stem diameter (14 ± 1 cm, 2.5 ± 0.9 mm), followed by the saplings growing under conditions of intermediate (10 ± 3 cm, 1.3 ± 0,8 mm) and severe water limitation (1 ± 1 cm, −0.4 ± 0.5 mm) (Fig. S3a,b, Table S3, *p* < 0.05). At the end of the main growing season, in autumn, the needle area of saplings growing under the control and intermediate water limitation treatments (1.5 ± 0.2 cm^2^ and 1.1 ± 0.1 cm^2^, respectively) was significantly larger than that of the saplings growing under severe water limitation (0.2 ± 0.1 cm^2^ Fig. S4a, Table S4, *p* < 0.001). The needle litter fall increased during the ^15^N experiment and was highest in autumn (Fig. S3c, Table S3, *p* < 0.001), as expected for evergreen pine species (e.g. Kinerson et al. 1974). The biomass of fine roots increased considerably over time for the saplings growing under the control and intermediate water limitation treatments (by 58% and 63%, respectively), while a negligible 25% increase was observed for the saplings growing under severe water limitation (Fig. S3d, Table S3). The A_net_ and g_s_ were observed to be significantly lower for the saplings growing under the severe treatment (4.18 ± 0.3 μmol m^−2^ s^−1^, 0.012 ± 0.01 mol m^−2^ s^−^) as compared to those growing under the control (9.7 ± 0.4 μmol m^−2^ s^−1^, 0.12 ± 0.01 mol m^−2^ s^−^) and intermediate (6.8 ± 0.4 μmol m^−2^ s^−1^, 0.06 ± 0.01 mol m^−2^ s^−^) treatments (Fig. S4b,c, Table S4, *p* < 0.001).

### Effects of water limitation on the cycling of nitrogen derived from decomposing needle litter

At the termination of the ^15^N experiment, part of the needle litter was still visible on the soil surface. As such, the percent of ^15^N label that was recovered in the soil and Scots pine saplings at the end of the experiment amounted to 22.1 ± 5.2% for the control treatment, 18.5 ± 3.0% for the intermediate water limitation treatment, and 10.4 ± 4.6% for the severe water limitation treatment. Additionally, some of the ^15^N label was likely lost through other non-investigated processes, such as gaseous losses or leaching. Below we report the results related to the influence of reduced water availability on the transfer of N derived from decomposing needle litter into the soil and Scots pine saplings.

***Soil:*** The application of the ^15^N enriched needle litter led to an increase in δ^15^N over the course of the experiment as compared to the soils where the natural abundance litter was added, for the entire investigated soil samples (0–20 cm, Fig. S5c, p < 0.05). The increase in δ^15^N between the soils exposed to either ^15^N enriched or natural abundance litter, overall led to positive Δ^15^N values, which were on average higher for the control and intermediate treatments Δ^15^N of 2.67 ± 0.60 ‰, and 1.88 ± 0.28 ‰ respectively, at the end of the experiment, Table 1) in comparison to the severe treatment (Δ^15^N of 1.32 ± 0.52 ‰).

**Table 1:** Seasonal change in Δ^15^N and percent of needle litter ^15^N recovered in the soil and Scots pine compartments throughout the ^15^N tracer experiment

Compartment		Δ^15^N [‰]				^15^N recovery [%]			
		Spring	Summer	Autumn		Spring	Summer	Autumn	
**Soil**	**Control**	0.66 ± 0.15	2.21 ± 0.49	2.67 ± 0.60	*	6.12 ± 2.49	18.45 ± 2.34	20.84 ± 4.99	*
	**Intermediate**	0.75 ± 0.36	1.97 ± 0.83	1.88 ± 0.28		6.79 ± 3.65	16.56 ± 6.65	16.55 ± 2.45	
	**Severe**	0.88 ± 0.19	0.89 ± 0.57	1.32 ± 0.52		8.20 ± 3.51	9.70 ± 4.10	10.34 ± 4.60	
**Needles**	**Control**	21.66 ± 9.75	18.56 ± 7.01	20.32 ± 7.10		0.93 ± 0.45	0.77 ± 0.32	0.63 ± 0.23	
	**Intermediate**	21.16 ± 8.70	22.55 ± 8.52	24.25 ± 8.93		0.01 ± 0.20	0.44 ± 0.22	0.38 ± 0.20	
	**Severe**	6.56 ± 3.38	7.06 ± 4.88	8.32 ± 4.41		0.01 ± 0.01	0.01 ± 0.01	0.01 ± 0.01	
**Fine roots**	**Control**	5.75 ± 2.55	3.53 ± 2.77	15.37 ± 6.82	*	0.11 ± 0.05	0.14 ± 0.10	0.62 ± 0.33	*
	**Intermediate**	4.28 ± 1.64	13.83 ± 5.11	40.11 ± 21.67	*	0.09 ± 0.05	0.58 ± 0.26	1.58 ± 0.76	*
	**Severe**	0.29 ± 0.81	0.44 ± 1.68	0.33 ± 0.71		0.01 ± 0.01	0.02 ± 0.02	0.02 ± 0.01	

Means ± standard errors (*n* = 3) are presented. Significant differences among sampling time points (seasons) across each irrigation treatment are expressed with an asterisk, *p* < 0.05 (*)

**Fig. 1:**
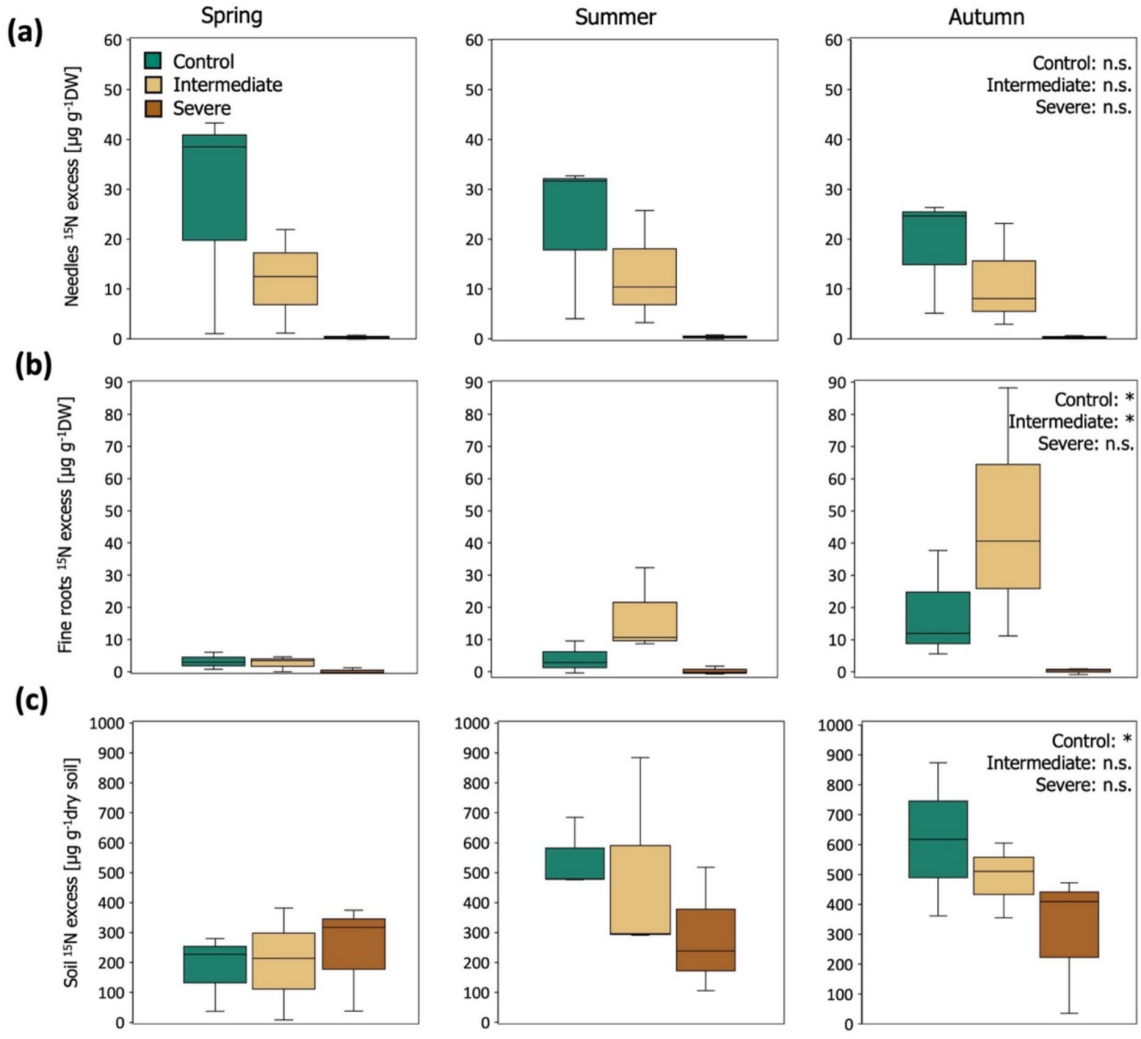
Seasonal variation of δ^15^N excess in the (a) new needles and (b) fine roots of Scots pine saplings (expressed as μg ^15^N excess in total dry weight (DW) of new needles or fine roots per sapling), and in the (c) soil (expressed as μg ^15^N excess in g dry soil in pot per mesocosm), throughout the ^15^N tracer experiment for the different irrigation treatments. In all panels, the lower and upper edges of the boxes mark the first and third quartiles of the data, and the median is represented by the line inside the boxes (n = 3). Significant differences among sampling time points (seasons) across each irrigation treatment, are expressed with an asterisk, *p* < 0.05 (*), *p* < 0.01 (**), *p* > 0.05 (n.s.)

The percent of ^15^N derived from the enriched needle litter which was recovered in the soil (sieved to < 2 mm) increased throughout the experiment in the soil of the control and intermediate treatments by an average of 240% and 144% respectively (Table 1). The recovered ^15^N in the soils exposed to severe water limitation only presented a minor average increase of 26% (Table 1). Seasonal differences in the soil ^15^N excess were observed (Fig. 1c). The highest soil ^15^N excess values were observed in summer (with an average of 519 ± 94 µg ^15^N g^−1^ dry soil, on a pot per mesocosm basis) and autumn (554 ± 79 µg ^15^N g^−1^ dry soil), and the lowest values in spring (191 ± 59 µg ^15^N g^−1^ dry soil) for the control and intermediate water limitation treatments. The ^15^N excess of the severely water-limited soil presented a smaller average variation throughout the study (279 ± 61 mg g^−1^ dry soil) (Fig. 1c).

***Scots pine saplings (new needles and fine roots):*** The saplings exposed to the ^15^N enriched label, presented higher δ^15^N values in their newly produced needles and fine roots when compared to the saplings grown under natural abundance conditions (Fig. S5a,b). This demonstrates that a substantial portion of the ^15^N label was incorporated in the Scots pine saplings after the start of the ^15^N tracer experiment, with the lowest increase in Δ^15^N observed for fine roots under the severe water limitation treatment (< 0.5 ‰ Δ^15^N, Table 1). During the ^15^N tracer experiment, the experimental treatments altered the ^15^N excess in the new needles and fine roots of the saplings (Fig. 1a, b). At the end of the ^15^N tracer experiment, in September, the new needles of the control saplings carried a markedly higher ^15^N excess (18 ± 7 µg^13^C g^−1^dry weight of new needles per sapling), than those of the saplings exposed to intermediate water deficit (11 ± 6 µg^13^C g^−1^ dry weight of new needles per sapling), and severe water deficit (0.3 ± 0.2 µg^13^C dry weight of new needles per sapling). The ^15^N excess in the fine roots was lowest for the saplings growing under severe water limitation (0.3 ± 0.5 µg^13^C g^−1^ dry weight of fine roots per sapling), followed by those of the control saplings (18 ± 10 µg^13^C g^−1^ dry weight of fine roots per sapling), and was observed to be comparatively higher for the saplings growing under intermediate water limitation (47 ± 23 µg^13^C g^−1^ dry weight of fine roots per sapling).

**Fig. 2:**
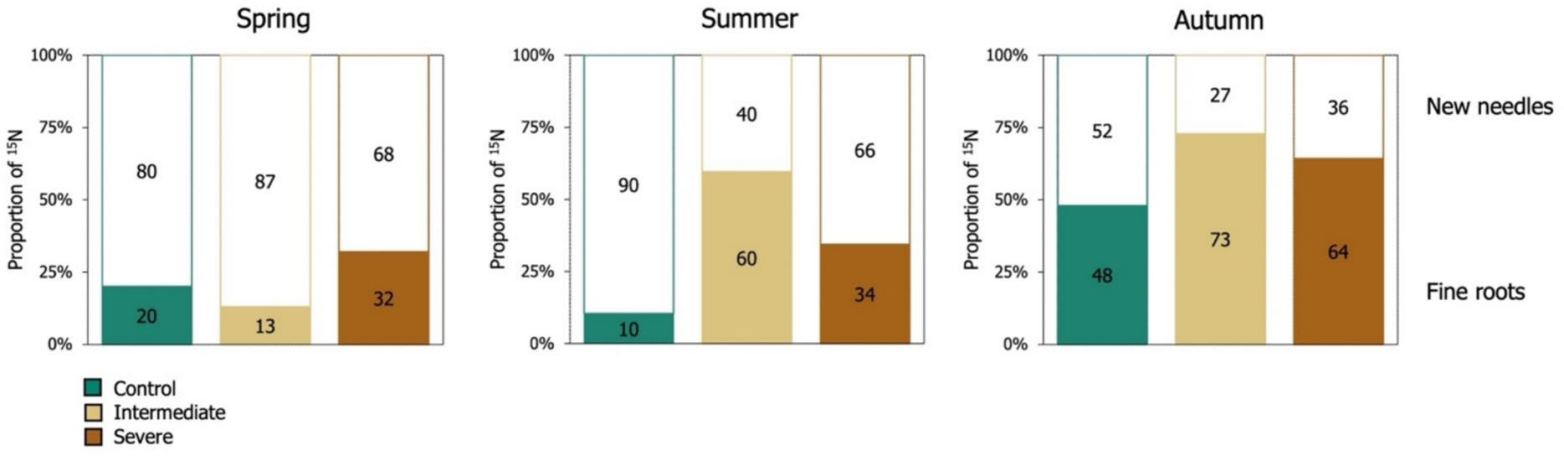
Seasonal changes in the relative fraction of ^15^N label allocated to new needles (empty boxes) and fine roots (coloured boxes) of Scots pine saplings, throughout the ^15^N tracer experiment for the different irrigation treatments

When considering the fraction of ^15^N label allocated to the investigated aboveground and belowground Scots pine compartments some seasonal patterns could also be observed. Most of the ^15^N label was observed to be allocated to the newly formed needles of the saplings in spring and summer (Fig. 2). Furthermore, the fraction of ^15^N label allocated to the fine roots presented an increase towards the end of the growing season, especially for the saplings growing under intermediate and severe water limitation. The highest fraction of ^15^N label allocated to fine roots was observed in autumn (Fig. 2).

**Fig. 3:**
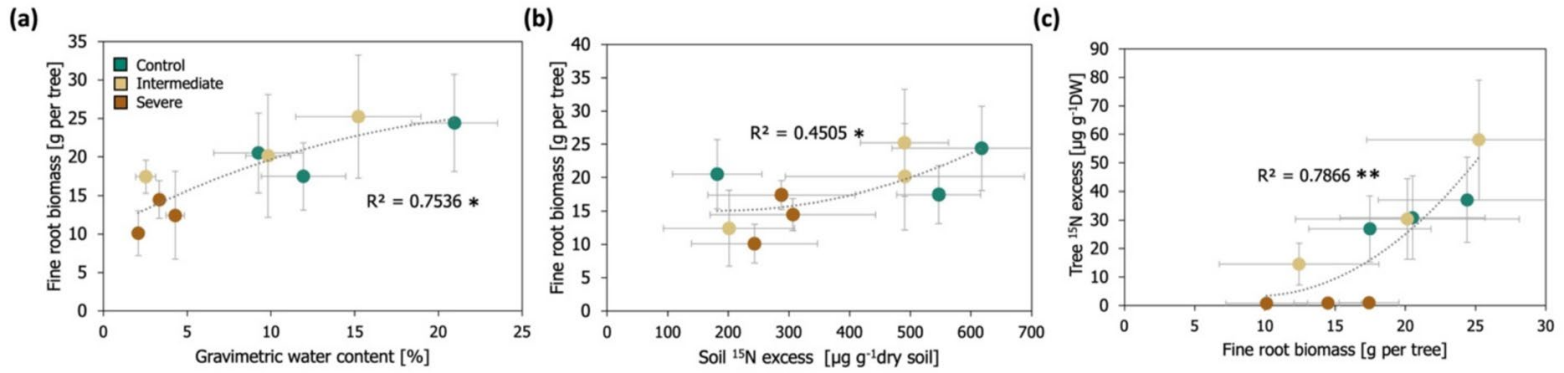
Relationship between **a**) the gravimetric water content of the soil and the fine root biomass of the saplings, **b**) the ^15^N excess in the soil (on a pot per mesocosm basis) and the fine root biomass of the saplings, **c**) the fine root biomass of the Scots pine saplings and the total amount of ^15^N label taken up by the saplings (new needles and fine roots), throughout the.^15^N tracer experiment for the different irrigation treatments. Each data point shows mean ± standard error (*n* = 3) for each sampling time point (season). Lines represent best fits to polynomial functions. The level of significance of the regressions is expressed with an asterisk, *p* < 0.05 (*), *p* < 0.01 (**)

Throughout the ^15^N tracer experiment, the fine root biomass of the saplings was positively related to the gravimetric water content of the soils (Fig. 3a, R^2^ = 0.75, *p* < 0.05) and the amount of ^15^N label in the soil (Fig. 3b, R^2^ = 0.45, *p* < 0.05). In turn, the total amount of ^15^N label measured in the saplings (^15^N excess in needles and roots) was positively related to the amount of fine root biomass of the saplings (Fig. 3c, R^2^ = 0.79, *p* < 0.01). The total ^15^N uptake of the saplings was instead not related to the ammonium (R^2^ = 0.35, *p* > 0.05), nitrate (R^2^ = 0.21, *p* > 0.05), or to total extractable N concentrations in soils (R^2^ = 0.44, *p* > 0.05).

### Effects of water limitation on soil chemical properties

Soil pH, ammonium, nitrate, total soil N concentrations, and soil C: N ratios were not influenced by the water limitation treatments during the ^15^N experiment (Fig. S6, Table S3). Ammonium and nitrate concentrations were highest in winter (2 ± 0.6 μgNH_4_^+^-N g^−1^dry soil, 12 ± 4 μgNO_3_^−^-N g^−1^dry soil, respectively, across all treatments), after which they declined and remained similar throughout spring (1.1 ± 0.2 μgNH_4_^+^-N g^−1^dry soil, 1.64 ± 0.46 μgNO_3_^−^-N g^−1^dry soil) to autumn (0.9 ± 0.2 μgNH_4_^+^-N g^−1^dry soil, 3.6 ± 0.85 μgNO_3_^−^-N g^−1^dry soil, Fig. S6c,d, Table S3, p < 0.01).

### Effects of water limitation on the soil microbial potential to cycle nitrogen

**Fig. 4:**
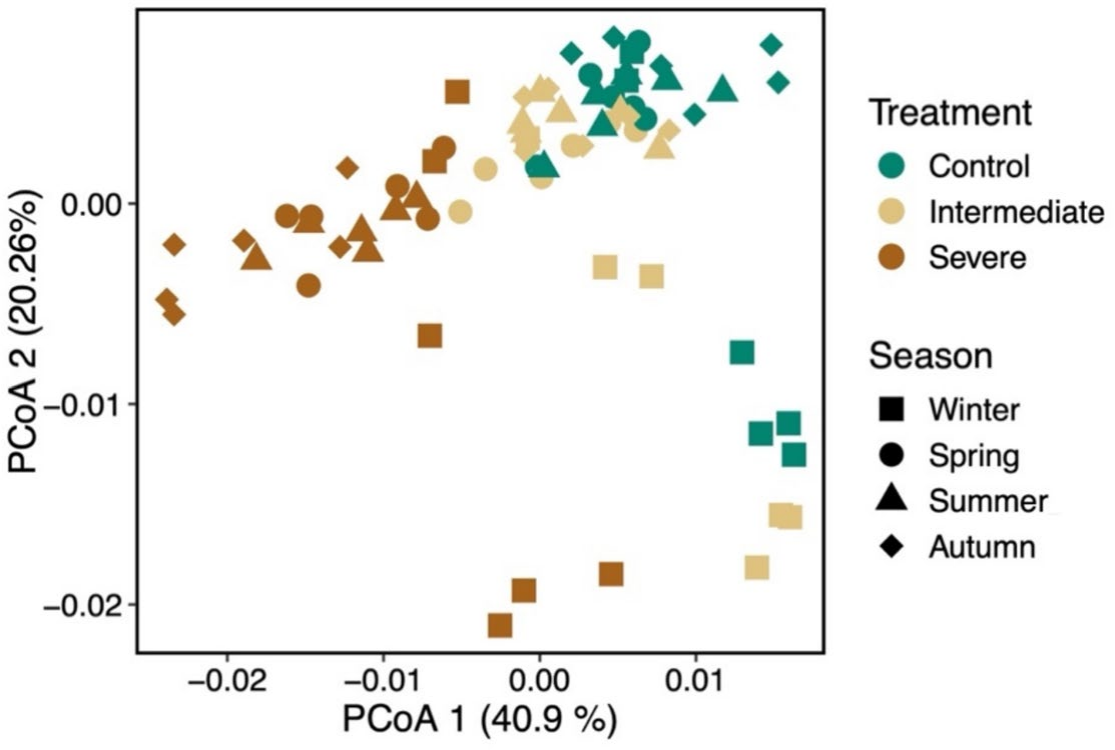
Principal coordinate analysis (PCoA) ordination showing shifts in the composition of nitrogen-cycling genes for the different irrigation treatments across all sampling time points (seasons)

The first axis of the PCoA identified irrigation treatment as the major determinant of differences in N-cycling gene composition, whereas the second axis described differences that were related to the sampling time point (season) (Fig. 4). PERMANOVA results further supported significant effects of irrigation treatment, sampling time point (season) and the interaction between treatment and season on N-cycling gene composition (Table 2).

**Table 2:** Effects of irrigation treatment (treatment, T), sampling time point (season, S), and their interaction (TxS) on the composition of genes associated with microbial nitrogen metabolism assessed by permutational analysis of variance (PERMANOVA)

	DF	R^2^	F	p
Treatment (T)	71	0.334	17.303	0.001
Season (S)	71	0.146	3.887	0.001**
(TxS)	71	0.535	6.281	0.001***

Values indicate the degrees of freedom (DF), F-ratio (F), the level of significance (p), and the explained variance (R^2^). Significant heterogeneities of variance assessed by permutational analysis of univariate dispersion (PERMDISP) are indicated with an asterisk, *p* < 0.01 (**), *p* < 0.001 (***)

The average dispersion (determined by PERMIDISP) was observed to be similar among the irrigation treatments (Table 2, *p* > 0.05), but was significantly greater in winter (0.013 ± 0.003) as compared to the other seasons (0.009 ± 0.004, Table 2, *p* < 0.01).

**Fig. 5:**
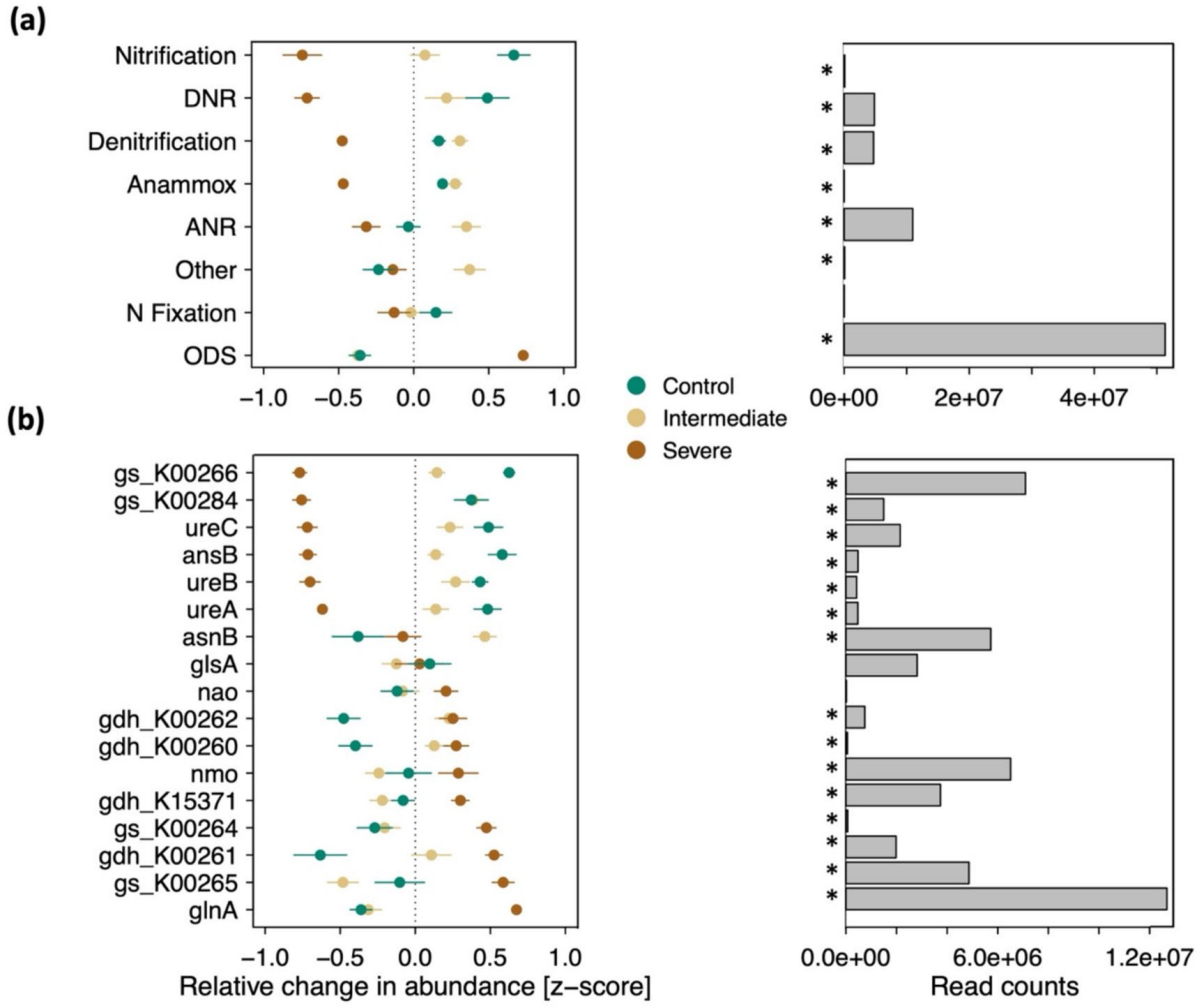
Relative change in abundance of (**a**) genes associated with different nitrogen cycling pathways, and (**b**) gene families potentially involved in organic degradation and synthesis, among the different irrigation treatments. The left panels represent the relative change in abundance (z-scores, means, and standard errors, *n* = 6) of (a) genes associated with nitrogen-specific cycling pathways in each treatment compared to the overall mean value across the entire dataset, and (b) gene families involved in organic degradation and synthesis in each treatment compared to the overall mean value across the entire dataset. The right panels represent (a) the number of assigned reads across all samples in each nitrogen cycling pathway, and (b) the number of assigned reads across all samples in each gene family involved in organic degradation and synthesis, (horizontal grey bars). Pathways that changed significantly are marked with the asterisk (*p* < 0.05). Dissimilatory nitrate reduction (DNR), assimilatory nitrate reduction (ANR), anaerobic ammonium oxidation (Anammox)**,** organic degradation and synthesis (ODS)

Most reads were annotated to the organic degradation and synthesis (ODS) pathway, followed by assimilatory nitrate reduction (ANR), dissimilatory nitrate reduction (DNR), and denitrification (Fig. 5a). Severe water limitation significantly increased the relative abundance of ODS genes, when compared to the control and the intermediate water limitation treatment. Comparatively, severe water limitation decreased the relative abundance of genes associated with ANR, DNR, and denitrification.

Within the ODS pathway, the prevalent gene family was associated with glutamine synthetase (*glnA*), for which the relative abundance increased under severe water limitation in comparison to the control and intermediate water limitation treatments (Fig. 5b). Other abundant gene families which increased under severe water limitation were related to nitronate monooxygenase (*nmo*), NADPH/NADH-dependent glutamate synthetase (*gs*_K00264), the large subunit of NADPH/NADH-dependent glutamate synthetase (*gs*_K00265), and glutamate dehydrogenase (*gdh*_K15371). Abundant gene families which instead declined under severe water limitation, included genes potentially encoding the small subunit of NADPH/NADH-dependent glutamate synthetase (*gs*_K00266) and ferredoxin-dependent glutamate synthase (*gs_K00284*). These patterns were observed to be consistent throughout all seasonal sampling times (Fig. S7).

## Discussion

**Fig. 6:**
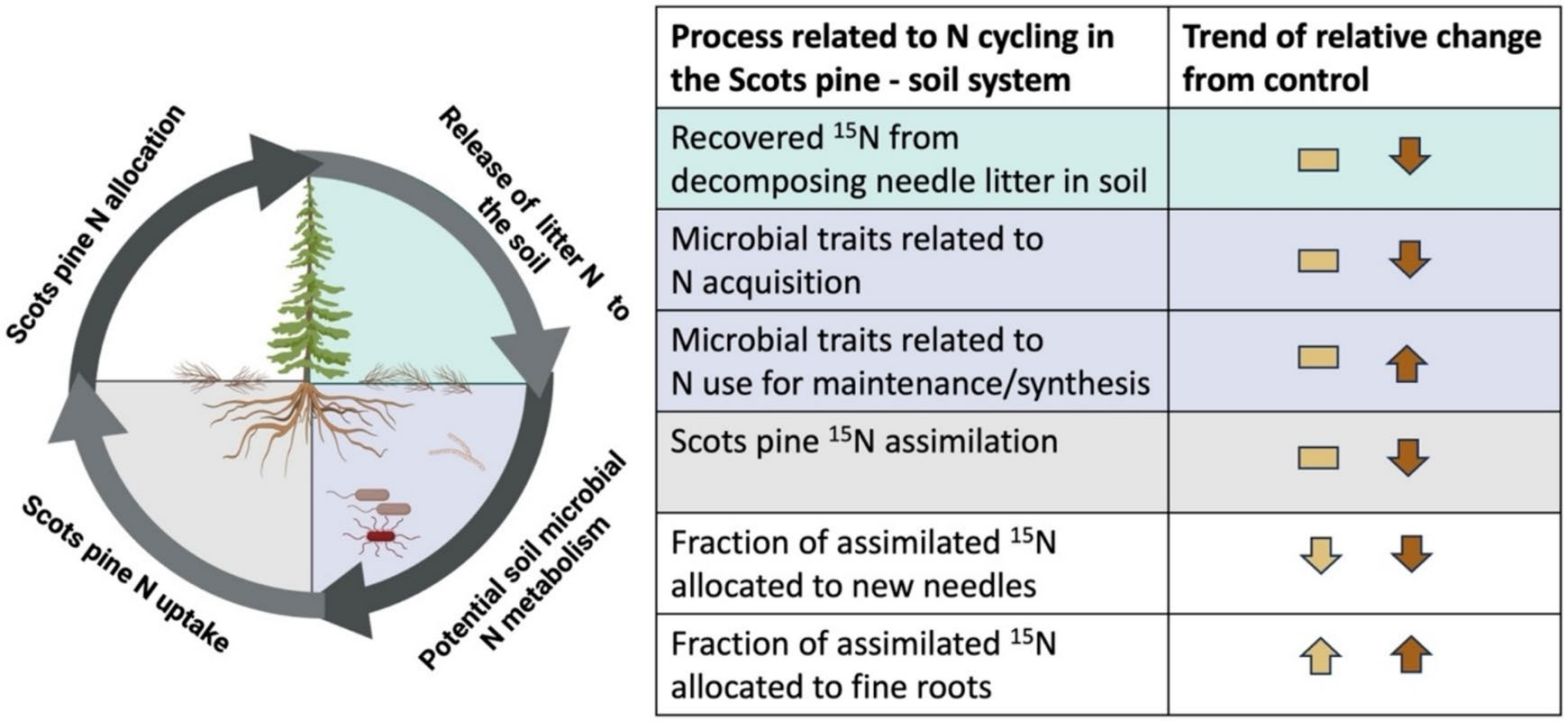
Summarizing scheme of the main findings of this study. The arrows represent the overall trend observed throughout the study for the intermediate water limitation treatment (dark yellow), and severe water limitation treatment (brown), when compared to the control treatment

Drought is expected to affect litter decomposition in terrestrial ecosystems by altering the biological activity of soil organisms. Moreover, lower soil moisture levels are also known to influence the physical dissolution or desorption of nitrogenous compounds from litter reducing the availability of plant-available N (Neff et al. 2003; Schaeffer et al. 2017; Schlesinger et al. 2016). Despite the significance of plant and microbial responses in predicting the impacts of drought on terrestrial N cycling, there is still limited direct evidence regarding the repercussions of progressive water loss on concomitant changes in the magnitude of aboveground and belowground N cycling processes. In this study, we addressed this challenge by combining ^15^N tracing techniques with metagenomic assessments of microbial genes involved in N cycling to evaluate how varying levels of soil water availability affected the fate of N derived from decomposing needle litter within a Scots pine saplings and forest soil mesocosm platform. Our findings demonstrated that the level of water limitation strongly influences the release of N from litter to the soil, the uptake of N by Scots pine saplings, the allocation of N into aboveground and belowground plant tissues, and the microbial functional potential associated with N cycling pathways in soils (Fig. 6).

### Influence of water limitation intensity on the recovery of needle-litter-derived nitrogen in the soil

The level of water limitation influenced the transfer of N derived from decomposing needle litter to the soil of the mesocosms (Fig. 1c, Table 1). In accordance with our first hypothesis, the amount of ^15^N label recovered in the soil at the end of the study was lowest under the severe water limitation treatment, followed by the intermediate and control treatments. However, contrary to our expectations, the amount of ^15^N label transferred from decomposing needle litter into the soil varied little under severe water limitation throughout the ^15^N experiment (by 26% on average). In contrast, it strongly increased over time under both the moderate water limitation and control treatments (by an average of 144% and 240% respectively, Fig. 1c, Table 1). The negligible change in the amount of ^15^N label recovered in the severely water-stressed soils during the study may be explained by several factors. First, a higher amount of ^15^N may have been leached from the needle litter into the soil immediately after the litter was added, rather than at later stages of decomposition during the ^15^N tracer experiment. An initial flush of N in the form of leachates is often observed during the early phases of plant litter decomposition (e.g. Berg and Staaf 1981). Second, although some nitrogenous compounds may have been leached or fragmented from the decomposing needle litter, most of these compounds may have been structurally complex and may have required high energy expenditures to be mineralized by soil microorganisms under conditions of water limitation (Hartmann et al. 2023; Neff et al. 2003; Su et al. 2023). Third, the observed decline in Scots pine development under severe water limitation may have impaired plant nutrient demands and reduced plant N uptake rates from the soil (Joseph et al. 2020). Plant N uptake demands may have been especially low in summer and autumn when the highest needle fall was observed for the severe treatment (Fig. S3). Nevertheless, our data showed that under severe water limitation, the total percentage of ^15^N label which was taken up by the Scots pine saplings was minimal, accounting for less than 1% of the needle-litter derived N recovered in the soil (Table 1). Consequently, seasonal changes in plant nutrient uptake are unlikely to fully explain the lack of variation in the amount of ^15^N label recovered in the severely stressed soils over the course of the experiment. It is more probable that severe water limitation may have disrupted the soil microbial metabolism involved in N cycling. Longer-term studies investigating the effect of drought on the incorporation of leaf litter with different quality (i.e. C: N ratio, lignin content) into different soil organic matter pools and microbial biomass would be needed to further test this hypothesis.

### Influence of water limitation intensity on the potential soil microbial N metabolism

Due to the small cell size and high surface-to-volume ratio that characterizes soil microorganisms, even small changes in soil water contents have the potential to instantaneously trigger changes in microbial metabolism. Nevertheless, our results showed that moderate water limitation barely affected the relative abundance of genes involved in soil N cycling pathways (Fig. 5). In contrast, severe water limitation strongly reshaped the microbial functional potential associated with N cycling pathways in soils. A reduction in soil water availability has been shown to select microbial populations with a genetic basis for stress tolerance (Hartmann et al. 2023; Jaeger et al. 2024; Malik et al. 2024), which we hypothesized could lead to a relative increase in the abundance of N-cycling genes potentially involved in maintenance and protection mechanisms, as opposed to genes associated with the uptake and release of N to the surrounding soil during mineralization and nitrification. In line with this hypothesis, we observed that genes annotated to the transformation of nitrogenous compounds to gain energy and N clearly declined under severe water deficit (i.e. nitrification, denitrification, dissimilatory nitrate reduction (DNR), and assimilatory nitrate reduction (ANR)), relative to the predominant organic degradation and synthesis pathway (ODS). Interestingly, all gene families involved in ODS became more distinct among the treatments (severe water limitation vs control and intermediate water limitation) over the course of the experiment (Fig. S7). These findings show that not only the structure of the soil microbiome, as previously observed in the same mesocosm platform (Jaeger et al. 2024), but also the metabolic potential of the microbiome, is altered by an extended period of water limitation due to continued adaptation of the communities to water-limiting conditions. In contrast, prokaryotic and fungal abundances in the mesocosms remained unaffected by the water limitation treatments (Jaeger et al. 2024).

In our study, the potential increase in the relative abundance of genes associated with ODS metabolism was primarily related to the enhanced regulation of the *glnA* gene, known to encode for the enzyme glutamine synthetase (Fig. 5b). Glutamine synthetase plays a central role in N metabolism and microbial protection against stresses like desiccation, as it converts glutamate and ammonia into glutamine (Forchhammer 2007). In turn, glutamine is required for the biosynthesis of various proteins. Our data further highlighted an increase in the relative abundance of genes encoding for enzymes involved in the synthesis of glutamate (NADPH/NADH-dependent *gs_K00264*) and α-ketoglutarate (glutamate dehydrogenase, *gdh_K15371*) under severe drought, as compared to the control and intermediate water limitation treatments (Fig. 5b). Glutamate and α-ketoglutarate are key metabolites in N assimilation and protein metabolism in microbes (Pierzynowski and Pierzynowska 2022; Walker and van der Donk 2016). Moreover, Csonka (1989) described glutamate as playing an essential role during drought periods in microbial osmoregulation and stress responses.

In bacteria, NADPH-dependent glutamate synthase consists of two non-identical subunits (a large subunit and a small subunit) (Pelanda et al. 1993). In yeast and fungi, NADH-dependent glutamate synthase consists of a single polypeptide chain with an N-terminal region similar to the bacterial large subunit, linked to a C-terminal region similar to the bacterial small subunit (Pire et al. 2014). Interestingly, in our study, under severe water limitation, the relative abundance of genes encoding the large chain of NADPH/NADH-dependent glutamate synthetase (*gs_K00265*) increased, while the relative abundance of genes encoding the small chain (*gs_K00266*) declined. Therefore, the inverse response of the large and small subunits of glutamate synthetase that we observed in this study suggests a decoupling of the two chains under severe drought conditions, potentially to reduce energy consumption during water deficit.

Another class of glutamate synthase is the ferredoxin-dependent glutamate synthase, which is commonly present in photosynthetic cells and cyanobacteria. In these organisms, ferredoxin plays a primary role in electron transfer during N fixation (Pire et al. 2014). In our study, we found that the relative abundance of genes encoding for ferredoxin-dependent glutamate synthase (*gs_K00284*) declined under severe water stress (Fig. 5b), corroborating the reduction in the relative abundance of N-fixing bacteria observed in the severely water-limited mesocosms observed in a previous study (Jaeger et al. 2024). These results indicate a microbial downregulation of ferredoxin during periods of prolonged water limitation, in line with previously observed declines in ferredoxin levels due to environmental stress (Lodeyro et al. 2012; Pierella Karlusich et al. 2014).

An additional notable increase in the relative abundance of genes involved in microbial N metabolism was observed for those encoding *nmo* (nitronate monooxygenase, Fig. 5b), an enzyme shown to help microorganisms defend themselves against toxic nitroalkanes by catalyzing their oxidative denitrification (Nguyen et al. 2022; Torres-Guzman et al. 2021).

 Although our results are a measure of change in the relative abundance of genes encoding for specific enzymatic transformation pathways, and do not directly reflect gene expression or enzymatic activity, they are an indication that when soil water becomes largely unavailable the functional capacity encoded by the soil microbiome is profoundly altered. In particular. they suggest an increase in the prevalence of microbial N-cycling genes potentially involved in mechanisms that protect against water stress, as opposed to genes associated with the uptake and release of N to the surrounding environment. Such trade-off, in turn, can feed back on organic N mineralization capabilities and decomposition rates of plant litter, supporting our observation of a reduced transfer of ^15^N label from decomposing needle-litter to severely dry soils.

### Influence of water limitation intensity on Scots pine N uptake and allocation

The acquisition of N by saplings from the soil primarily depends on nutrient availability at the root surface and the root uptake surface area (Joseph et al. 2020). Our results did not show significant changes in soil ammonium, nitrate, and total N concentrations among the three irrigation treatments during the ^15^N tracer experiment (Fig. S6, Table S3), probably due to the concurrent reductions in N uptake by the saplings and in the slower decomposition and soil mineralization rates under conditions of severe water limitation. Nevertheless, as we had hypothesized, the levels of water limitation strongly shaped the root biomass growth of the saplings, and thus, the active surface area for nutrient uptake. We observed that living root biomass increased throughout the experiment in the saplings growing under the control and intermediate water limitation treatments but not in saplings growing under severe water limitation (Fig. S3d, Table S3). Our results also indicated that the fine root biomass of the saplings was closely related to the total amount of ^15^N label which was taken up by the plants (Fig. 3c) with the highest ^15^N excess in fine roots observed for the trees growing under intermediate water limitation (Fig. 1). These findings confirm that fine roots are crucial for tree nutrient uptake and that severe levels of drought can significantly impede root growth and fine root N uptake (Brunner et al. 2015; de Kroon and Mommer 2006; Joseph et al. 2020). It is important to note that the fine root biomass assessments in this study were conducted during the second growing season of the saplings in the mesocosms, which differed significantly from the fine root biomass assessment made during the first growing season of the saplings. During the first growing season, root biomass was not observed to significantly differ among the irrigation treatments (Solly et al. 2023). This suggests that the fine root growth of Scots pine trees is more likely to be impaired by prolonged periods of intense water limitation rather than by short episodes of drought.

In relative terms, saplings growing under water-limited conditions were found to allocate a higher fraction of ^15^N label to their fine root systems compared to new needles, in contrast to control saplings (Fig. 2). This suggests that to compensate for reduced ion mobility under soil water deficit, plants may allocate more N to their root systems to support their physiology and enhance drought resilience (Gessler et al. 2017; Poorter et al. 2012). For example, an increased N allocation to fine roots in the form of osmoprotective amino acids has been suggested to support drought tolerance in other tree species (Fotelli et al. 2002).

Some seasonal dynamics could also be observed. At the beginning of the growing season of the saplings, in spring and summer, most of the ^l5^N label taken up by the saplings was allocated to sustain the growth of new needles, and only a smaller amount of ^15^N label was partitioned to fine roots (Fig. 2). Instead, in autumn, a larger amount of ^15^N was allocated to fine roots, especially under the intermediate water limitation treatment, where the highest ^15^N excess was observed (Fig. 1b). The increase in the amount of ^15^N label recovered in fine roots towards the end of the growing season likely reflects an accumulation of N to support the growth of the saplings, either during the following spring or upon the rewetting of the soils. During spring, when the N required for new root and foliage growth exceeds the amount of recently absorbed N, trees are believed to rely heavily on stored N (Millard and Proe 1992). This allows trees to temporally uncouple N demand and external N supply. In contrast, during summer, N demands are primarily realized through root N uptake from the soil (Millard and Proe 1992). Therefore, any reduction in N uptake during drought periods could have a strong negative impact on tree function (Gessler et al. 2017).

We cannot rule out that higher amounts of ^15^N label are allocated to the plant tissues growing most during a specific period (e.g. new needles in spring and fine roots in autumn). A higher sampling frequency over shorter time intervals may help future assessments to better link changes in the growth of trees to the uptake and redistribution of N within plants*.* However, our data suggest that seasonal growth patterns are unlikely to explain the full extent of the observed ^15^N label allocation patterns. For instance, we found that under severe water limitation, the saplings barely increased their fine root growth after winter. Moreover, control and moderately stressed saplings mainly grew their fine roots in summer, not in autumn, when the largest increase in ^15^N allocation to roots was observed in the control group.

We can further not exclude the possibility that water limitation may have affected the efficiency of plant N uptake. However, in a recent study which linked N uptake to C allocation belowground in drought-exposed tree saplings (Joseph et al. 2020), the root uptake capacity of Scots pine saplings did not appear to be affected by drought, as long as the energy demands for nutrient uptake were met by sufficient carbohydrates in the root systems. In our study, the soil moisture in the mesocosms under severe water limitation was kept at a level that allowed the saplings to receive a minimum amount of water to remain vital, ensuring that C transport through the phloem could continue, albeit at a reduced pace (Solly et al. 2023). Additionally, a previous study in the same mesocosms showed that newly fixed C was accumulated in the root systems of severely water-stressed saplings (Solly et al. 2023), supporting the idea that an impaired root uptake capacity due to water stress was at most a minor contributor to the altered ^15^N label allocation patterns. It is more plausible that N partitioning was temporally coordinated with seasonal and drought-induced fluctuations in plant physiology and N demands, optimizing the cost–benefit trade-offs between plant N acquisition and conservation (Zhao et al. 2024).

## Conclusions

The results of this study highlight that the level of soil water differently affects the uptake of soil N by Scots pine saplings and the N-cycling potential of the soil microbiome. While under intermediate water limitation, Scots pine saplings may acclimate and potentially increase N uptake to support their metabolism, more severe levels of water limitation strongly reduce Scots pine N uptake. In general, our findings indicate that water limitation increases the fraction of newly acquired N allocated to fine root systems of Scots pine saplings, likely reflecting an accumulation of N to support growth during the following growing season or upon rewetting of the soils. In our study, the potential of the soil microbiome to cycle N was observed to be only moderately affected by intermediate water limitation. In contrast, severe levels of water deficit increased the proportion of microbial N-cycling genes potentially involved in mechanisms protecting against water stress, comparatively to genes associated with the uptake and release of N to the surrounding soil. The highlighted tradeoffs between plant and microbial tolerance and N resource acquisition and decomposition suggest that long-lasting episodes of drought will slow down the cycling of N due to changes in aboveground and belowground N metabolism. This work opens new insights into the mechanistic understanding of the processes involved in the initial transfer of N derived from decomposing plant litter to aboveground and belowground components under different intensities of experimental water limitation, which may help to improve biogeochemical model predictions of N cycling at the plant and soil interface under future global change scenarios. Future research using experimental settings or studies in the field should explore the long-term effects of water limitation on the release of N from decomposing plant litter of different quality in forest soils.

## Supplementary Information

Below is the link to the electronic supplementary material.

Supplementary file1 (XLSX 31 KB)

Supplementary file2 (XLSX 18 KB)

Supplementary file3 (DOCX 2.51 MB)

## Data Availability

The raw reads of the soil microbial metagenome are deposited in NCBI's sequence read archive under the BioProject link: https://www.ncbi.nlm.nih.gov/bioproject/PRJNA1007885. The plant and soil data that support the findings of this study are provided in the Supplementary Information file.
