# Supplementary material for: Soil water limitation intensity alters nitrogen cycling at the plant-soil interface in Scots pine mesocosms
Source: Plant Soil. 2025 Aug 26;516(1):705–23. doi: 10.1007/s11104-025-07758-z (PMC12764678; doi:10.1007/s11104-025-07758-z)
Supplement: Supplementary file 3 — Supplementary file3 (DOCX 2.51 MB) [file 11104_2025_7758_MOESM3_ESM.docx]

**Supporting information**

Article title: Soil water limitation intensity alters nitrogen cycling at the plant-soil interface in Scots pine mesocosms

Authors: Emily F. Solly, Astrid C. H. Jaeger, Matti Barthel, Johan Six,

Ralf C. Mueller, Martin Hartmann

Author for correspondence:

*Emily F. Solly*

*Email: emily.solly@ufz.de*

The following Supporting Information is available for this manuscript:

**Fig. S1:** Scheme summarizing the preparation of the ^15^N-enriched, and natural abundance needle litter, their addition to the mesocosms, and pictures of the mesocosms.

**Fig. S2:** Volumetric water content in % measured continuously in each of the 18 mesocosms after the start of the irrigation treatments.

**Fig. S3:** Seasonal variation in the height increment, radial growth, needle litterfall, and fine root biomass of Scots pine saplings throughout the study, under different irrigation treatments.

**Fig. S4:** Differences in the needle area, light-saturated photosynthesis, and stomata conductance of Scots pine saplings among the irrigation treatments.

**Fig. S5:** Seasonal variation of δ^15^N in the new needles and fine roots of Scots pine saplings, and in the soil, throughout the ^15^N tracer experiment, under different irrigation treatments.

**Fig. S6:** Seasonal changes in the pH, total nitrogen, ammonium concentrations, and nitrate concentrations of the soil, throughout the study for the different irrigation treatments.

**Fig. S7:** Visualization of the relative change in abundance of the gene families involved in organic degradation and synthesis among the different irrigation treatments for each seasonal sampling time.

**Table S1:** Seasonal greenhouse air temperature and humidity during the course of the mesocosm experiment.

**Table S2:** Seasonal values of soil gravimetric water content (GWC [%]) for each irrigation treatment.

**Table S3:** Effects of irrigation treatment (treatment, T), sampling time point (season, S) their interaction (TxS) on soil physicochemical parameters, and Scots pine parameters, tested with linear mixed effect models (lme).

**Table S4:** Effects of irrigation treatment (treatment, T) on Scots pine sapling needle area and gas exchange parameters, tested with linear mixed effect models (lme).

**Methods S1:** Additional details on leaf gas exchange measurements.

**Methods S2:** Additional details on the total dry weight assessments of the Scots pine saplings.

**Methods S3:** Additional information on sequencing data processing.

**Dataset S1:** Soil gravimetric water content (GWC), Scots pine sapling development and gas exchange, soil properties, and sapling total N concentrations and C : N ratios. Separate file (.xlsx).

**Dataset S2:** Scots pine sapling and soil N isotopic composition. Separate file (.xlsx).

**Figure S1:** (a) Scheme summarizing the preparation of the ^15^N-enriched, and natural abundance needle litter, and their addition on the surface of the soil in the mesocosms. (b) Mesocosms supplied with ^15^N-enriched needle litter were separated in a different greenhouse (purple) from the mesocosms supplied with natural abundance needle litter (white) to avoid cross-contamination after labeling with ^15^N. (c) Pictures of the mesocosms taken in spring, where the color of the labels indicates the different irrigation treatments: control (green), intermediate water limitation (yellow), severe water limitation (red).

**
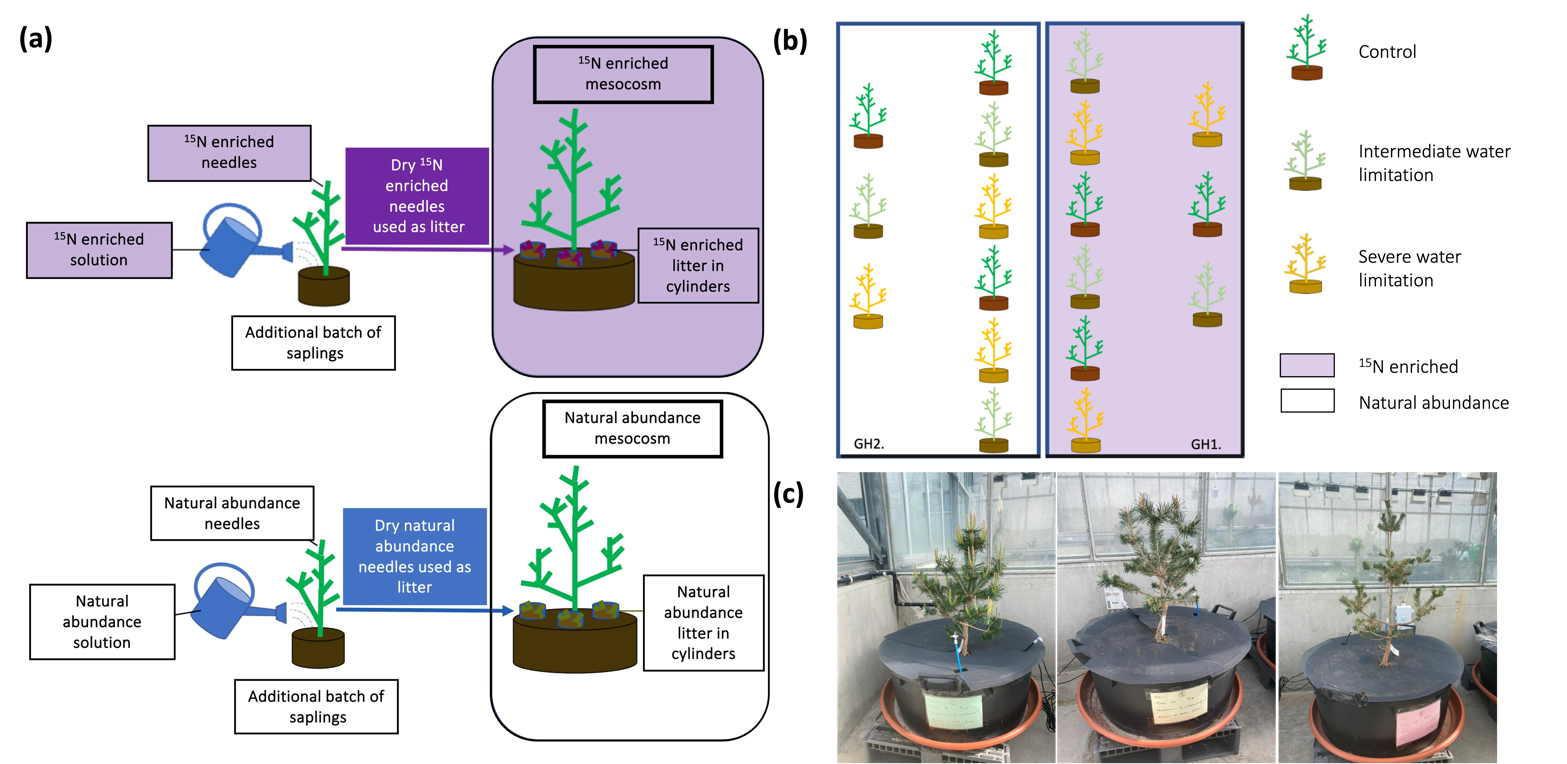
**

**Figure S2:** Volumetric water content in % measured continuously in each of the 18 mesocosms after the start of the irrigation treatments. The lines represent the hourly volumetric water content data averaged across mesocosms and the shaded bands show the standard error (n=6). Control (green), intermediate water limitation (dark yellow), severe water limitation (brown). The arrows illustrate the start of the irrigation treatments and the start of the ^15^N tracer experiment.

**Figure S3:** Seasonal changes in the (a) height increment, (b) radial increment, (c) needle litterfall, and (d) fine root biomass of Scots pine saplings, throughout the study under the different irrigation treatments. In all panels, the means ± standard errors (n= 6) are presented.


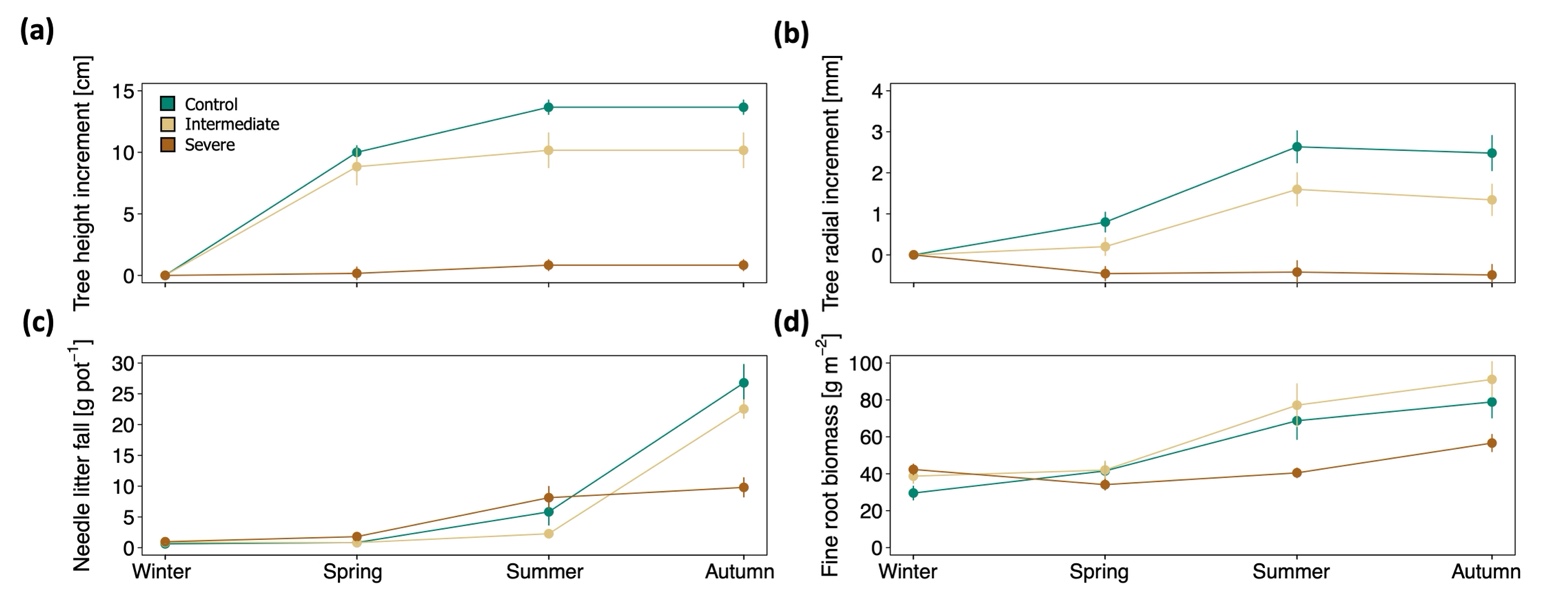


**Figure S4:** Differences among irrigation treatments, in Scots pine saplings (a) needle area (normalized per needle) at the end of the ^15^N tracer experiment, (b) light-saturated photosynthesis (A_net_) at the beginning of the ^15^N tracer experiment, and (c) stomata conductance (g_s_) at the beginning of the ^15^N tracer experiment. In all panels, the lower and upper edge of the boxes mark the first and third quartiles of the data, and the median is represented by the line inside the boxes (n = 6).

**
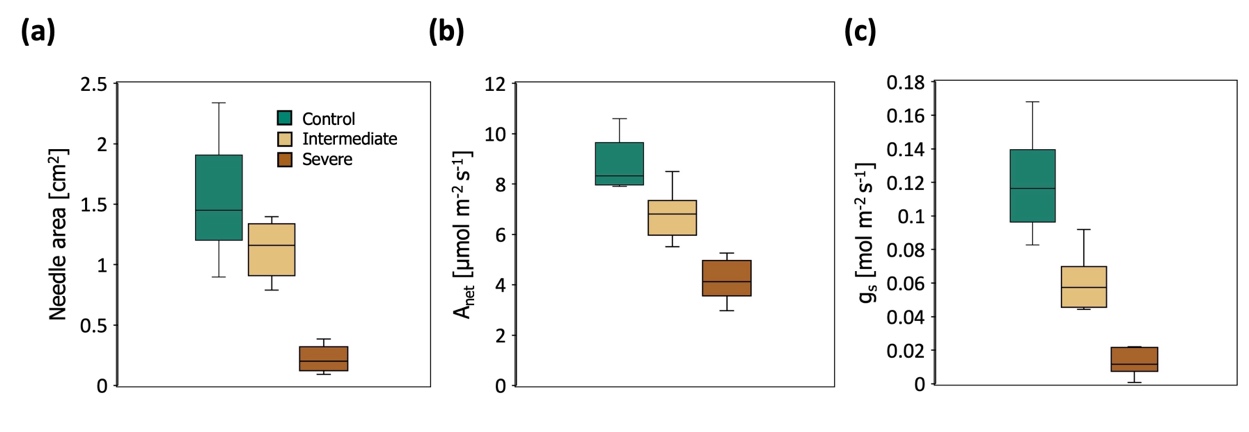
**

**Figure S5:** Seasonal variation of δ^15^N in the (a) new needles and (b) fine roots of Scots pine saplings, and in the (c) soil, throughout the ^15^N tracer experiment for the different irrigation treatments, in mesocosms where ^15^N enriched (filled boxes) and natural abundance (empty boxes) needle litter was added. In all panels, the lower and upper edge of the boxes mark the first and third quartiles of the data, and the median is represented by the line inside the boxes (n = 6). Significant differences between ^15^N enriched and natural abundance new needles, fine roots and soils for each irrigation treatment throughout all sampling time points (seasons) are expressed with an asterisk, p < 0.05 (*), p < 0.01 (**), p < 0.001 (***), p > 0.05 (n.s.).

**
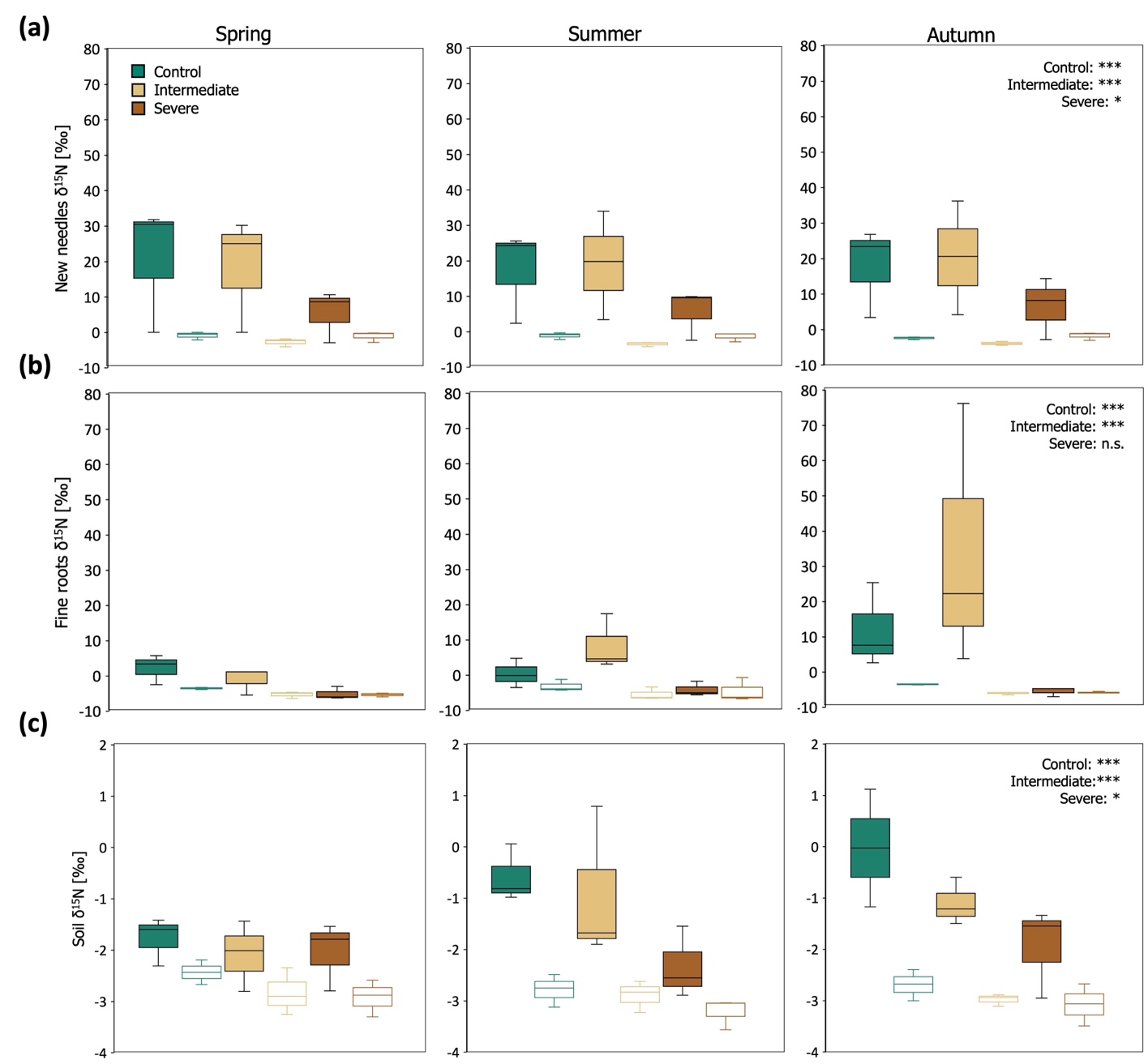
**

**Figure S6:** Seasonal changes in the (a) pH, (b) total nitrogen, (c) ammonium concentrations, and d) nitrate concentrations of the soil, throughout the study for the different irrigation treatments. In all panels, the means ± standard errors (n= 6) are presented. **
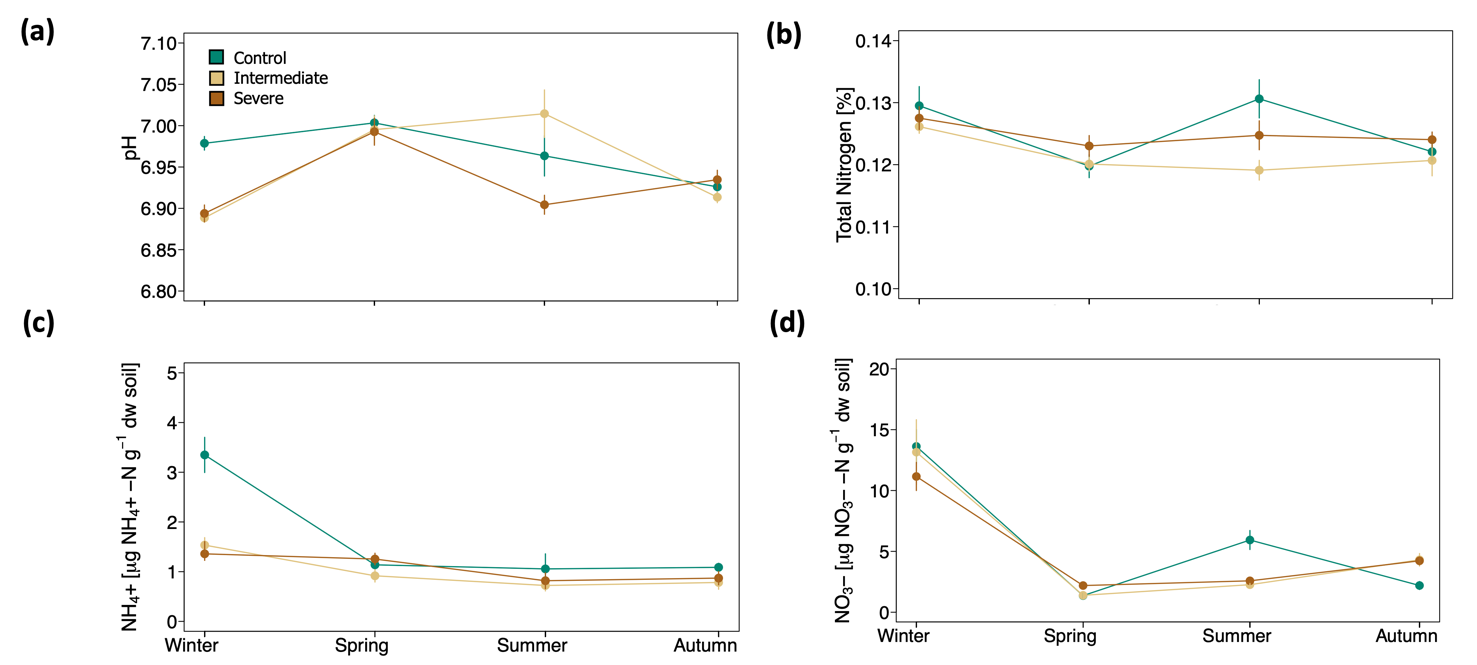
**

**Figure S7:** Visualization of the relative change in abundance of the gene families involved in organic degradation and synthesis among the different irrigation treatments for each sampling time point (season). The panels represent the relative change in abundance (z-scores, means ± standard errors, n = 6) of gene families in each treatment compared to the overall mean value of the entire datasets.

**
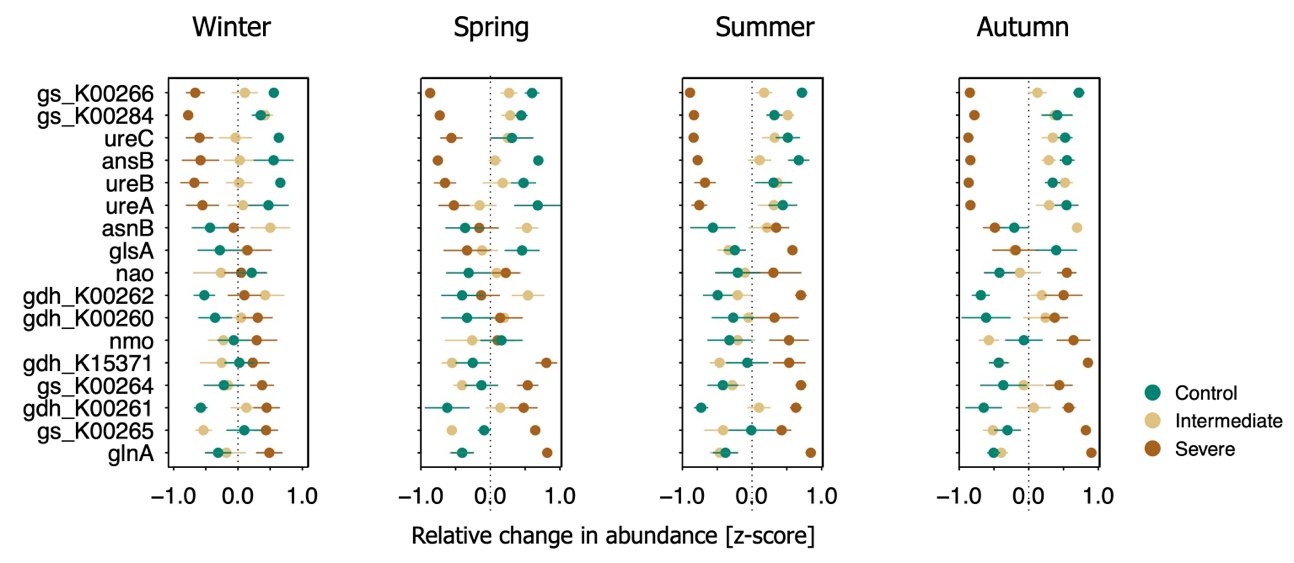
**

**Table S1:** Seasonal greenhouse (GH) air temperature and humidity. Means as well as minimum and maximum values (min – max) are presented.

| Parameter | **Winter** | **Spring** | **Summer** | **Autumn** |
| --- | --- | --- | --- | --- |
| GH air temp. [°C] | **10** (8 – 23) | **16.0** (11.5 – 30.0) | **22.6** (15.4 – 30.4) | **15.7** (9.5 – 26.4) |
| GH humidity [%] | **50** (8 – 79) | **41** (10 – 67) | **62** (22 –95) | **60** (36 – 93) |

**Table S2:** Seasonal values of soil gravimetric water content (GWC [%]) for each irrigation treatment. Means ± standard errors (n = 6) are presented.

| Treatment | **Winter** | **Spring** | **Summer** | **Autumn** |
| --- | --- | --- | --- | --- |
| Control | 33.9 ± 0.8 | 10.2 ± 1.5 | 12.3 ± 1.5 | 21.4 ± 1.3 |
| Intermediate | 13.8 ± 0.7 | 3.8 ± 0.3 | 7.8 ± 1.2 | 12.3 ± 2.1 |
| Severe | 4.7 ± 0.4 | 2.8 ± 1.2 | 2.3 ± 0.3 | 3.4 ± 0.2 |

**Table S3:** Effects of irrigation treatment (treatment, T), sampling time point (season, S) their interaction (TxS) on soil physicochemical parameters, and Scots pine sapling parameters, tested with linear mixed effect models (lme) and displayed with the numerator degrees of freedom (nDF), denominator degrees of freedom (dDF), F-ratio (F) and level of significance p (p-value). Significant results are indicated with bold numbers.

| **Parameter** | **Treatment (T)** | | | | **Season (S)** | | | | **T x S** | | | |
| --- | --- | --- | --- | --- | --- | --- | --- | --- | --- | --- | --- | --- |
|  | **nDF** | **dDF** | **F** | **p** | **nDF** | **dDF** | **F** | **p** | **nDF** | **dDF** | **F** | **p** |
| **GWC** | 2 | 14 | 84.44 | **<0.001** | 3 | 45 | 41.18 | **<0.001** | 6 | 45 | 1.29 | 0.281 |
| **pH** | 2 | 14 | 1.54 | 0.249 | 3 | 45 | 4.84 | **0.005** | 6 | 45 | 1.96 | 0.927 |
| **Soil ammonium** | 2 | 14 | 3.30 | 0.0668 | 3 | 45 | 6.57 | **0.001** | 6 | 45 | 0.98 | 0.452 |
| **Soil nitrate** | 2 | 14 | 0.63 | 0.548 | 3 | 45 | 41.99 | **<0.001** | 6 | 45 | 3.902 | 0.003 |
| **Soil total N** | 2 | 14 | 0.69 | 0.515 | 3 | 45 | 1.89 | 1.144 | 6 | 45 | 0.545 | 0.770 |
| **Soil C:N ratio** | 2 | 14 | 0.28 | 0.763 | 3 | 45 | 1.32 | 0.279 | 6 | 45 | 0.74 | 0.624 |
| **Sapling height growth** | 2 | 14 | 13.20 | **<0.001** | 3 | 45 | 44.88 | **<0.001** | 6 | 45 | 9.99 | **<0.001** |
| **Sapling radial growth** | 2 | 14 | 7.07 | **0.007** | 3 | 45 | 7.74 | **0.001** | 6 | 45 | 3.49 | **0.006** |
| **Needle litter fall** | 2 | 14 | 0.30 | 0.746 | 3 | 45 | 63.61 | **<0.001** | 6 | 45 | 2.76 | **0.023** |
| **Fine root biomass** | 2 | 14 | 0.59 | 0.566 | 3 | 45 | 15.48 | **<0.001** | 6 | 45 | 1.81 | 0.118 |
| **New needle total N** | 2 | 14 | 5.45 | **0.018** | 3 | 45 | 0.32 | 0.730 | 6 | 45 | 3.73 | **0.014** |
| **New needle C:N ratio** | 2 | 14 | 4.15 | **0.038** | 3 | 45 | 0.05 | 0.954 | 6 | 45 | 3.52 | **0.018** |
| **Fine root total N** | 2 | 14 | 1.23 | 0.319 | 3 | 45 | 6.58 | **0.004** | 6 | 45 | 0.43 | 0.788 |
| **Fine root C:N ratio** | 2 | 14 | 1.38 | 0.283 | 3 | 45 | 4.21 | **0.024** | 6 | 45 | 0.55 | 0.698 |

**Table S4:** Effects of irrigation treatment (Treatment, T), on Scots pine sapling needle area and gas exchange parameters, tested with linear mixed effect models (lme) and displayed with the numerator degrees of freedom (nDF), denominator degrees of freedom (dDF), F-ratio (F) and level of significance p (p-value). Significant results are indicated with bold numbers.

| **Parameter** | **Treatment (T)** | | | |
| --- | --- | --- | --- | --- |
|  | **nDF** | **dDF** | **F** | **p** |
| **Needle area** | 2 | 13 | 47.41 | **<0.001** |
| **Photosynthesis (A_net_)** | 2 | 13 | 76.32 | **<0.001** |
| **Stomatal conductance (g_s_)** | 2 | 13 | 52.02 | **<0.001** |

**Methods S1: Additional details on leaf gas exchange measurements**

25 south exposed needles were enclosed in the 2x3 cm chamber, and Anet and gs were measured under 400 μmol mol-1 CO2, 1000 PAR, local humidity and temperature, and a stomatal ratio of 1. The leaf area of the needles was also determined at the end of the growing season by randomly collecting 30 - 40 needles throughout the whole crown of the saplings and scanning them with a flatbed scanner (EPSON Expression 11000XL, EPSON, Suwa, Nagano, Japan). The scanned images were analyzed with the WinRHIZO program (version 2013, Regent Instruments Inc., Chemin Sainte-Foy, Quebec, Canada) to determine the leaf area of the needles (Albaugh et al. 2020), which was normalized per needle.

**Methods S2: Additional details on Scots pine saplings total dry weight assessments**

The total needle biomass at each sampling time was obtained for each individual sapling based on oven-dried needle weight and the estimation of the total number of needles on the branches of the saplings. The total biomass of living fine roots and soil (< 2 mm) in the mesocosms was estimated by considering the dry weight of living fine roots (< 2 mm) recovered from the collected soil cores and extrapolating that amount to the whole pot for each mesocosm at every sampling time. To ensure that the living fine root biomass values at each sampling time were plausible, the values were compared to those determined during the final harvest of the saplings at the end of the experiment. This comparison showed a variation of 8 ± 4% between the living fine root biomass that was estimated and determined at the final harvest.

**Methods S3: Additional information on sequencing data processing**

All samples were processed individually. First, the raw sequence data was quality-checked with FastQC v0.11.9 (Andrews et al. 2012), and Illumina adapters and poly-G/N tails were trimmed with fastp v0.22.0 (Chen et al. 2018). Low-complexity reads were filtered with PRINSEQ v0.20.4 (Schmieder and Edwards 2011) and phi X 174 DNA sequences were removed with BOWTIE2 v2.5.0 (Langmead and Salzberg 2012) with the reference sequence downloaded from Illumina (<https://support.illumina.com/sequencing/sequencing_software/igenome.html>, PhiX Illumina RTA, download: 11.08.2022). The resulting reads were quality-filtered using VSEARCH v2.22.1 and dereplicated with czid-dedup v0.1.0 (https://github.com/chanzuckerberg/czid-dedup). Eventually, 90.8% (3.796 Tb) of the initial sequencing yield passed pre-processing.

The pre-processed reads were assembled into contigs using MEGAHIT v1.2.9 (Li et al. 2015) with the preset ‘meta-large’. The average length of the assemblies was 3.45 Gb (SD = 0.62 Gb) with a mean contig N50 of 549 bp (SD = 9.9 bp; minimum contig length set to 200 bp). Putative open reading frames were predicted by PRODIGAL v2.6.3 (Hyatt et al. 2010) and reported as protein-coding nucleotide (nt) sequences and translated amino acid (aa) sequences. In total, 1.179 G genes were predicted with a total nt length of 449 Gb to which pre-processed reads were mapped with BOWTIE2. Afterwards, mapping statistics were extracted using SAMTOOLS v1.16.1 (Danecek et al. 2021; Li et al. 2009) and appended to the FASTA headers of the aa sequences of predicted genes.

Genes associated with N metabolism were annotated using the NCyc database (Tu et al. 2018). Specifically, the NCyc (100_2019Jul), sequence data was downloaded and prepared for DIAMOND alignments. Predicted genes were then aligned to these references using DIAMOND with the E-value set to 10^-5^.

**References:**

Albaugh TJ, Maier CA, Campoe OC, Yáñez MA, Carbaugh ED, Carter DR, Cook RL, Rubilar RA, Fox TR (2020) Crown architecture, crown leaf area distribution, and individual tree growth efficiency vary across site, genetic entry, and planting density. Trees 34: 73-88. doi: 10.1007/s00468-019-01898-3.

Andrews S, Krueger F, Segonds-Pichon A, Biggins L, Krueger C, Wingett S (2012) FastQC. <https://www.bioinformatics.babraham.ac.uk/projects/fastqc/> (accessed 9.12.2023).

Chen S, Zhou Y, Chen Y, Gu J (2018) fastp: an ultra-fast all-in-one FASTQ preprocessor. Bioinformatics 34: i884-i890. doi: 10.1093/bioinformatics/bty560.

Danecek P, Bonfield JK, Liddle J, Marshall J, Ohan V, Pollard MO, Whitwham A, Keane T, McCarthy SA, Davies RM, Li H (2021) Twelve years of SAMtools and BCFtools. GigaScience 10. doi: 10.1093/gigascience/giab008.

Hyatt D, Chen G-L, LoCascio PF, Land ML, Larimer FW, Hauser LJ (2010) Prodigal: prokaryotic gene recognition and translation initiation site identification. BMC Bioinformatics 11: 119. doi: 10.1186/1471-2105-11-119.

Langmead B, Salzberg SL (2012) Fast gapped-read alignment with Bowtie 2. Nature Methods 9: 357-359. doi: 10.1038/nmeth.1923.

Li D, Liu C-M, Luo R, Sadakane K, Lam T-W (2015) MEGAHIT: an ultra-fast single-node solution for large and complex metagenomics assembly via succinct de Bruijn graph. Bioinformatics 31: 1674-1676. doi: 10.1093/bioinformatics/btv033.

Li H, Handsaker B, Wysoker A, Fennell T, Ruan J, Homer N, Marth G, Abecasis G, Durbin R, Subgroup GPDP (2009) The Sequence Alignment/Map format and SAMtools. Bioinformatics 25: 2078-2079. doi: 10.1093/bioinformatics/btp352.

Schmieder R, Edwards R (2011) Quality control and preprocessing of metagenomic datasets. Bioinformatics 27: 863-864. doi: 10.1093/bioinformatics/btr026.

Tu Q, Lin L, Cheng L, Deng Y, He Z (2018) NCycDB: a curated integrative database for fast and accurate metagenomic profiling of nitrogen cycling genes. Bioinformatics 35: 1040-1048. doi: 10.1093/bioinformatics/bty741.
